# Oxidized Spiro-OMeTAD: Investigation of Stability in Contact with Various Perovskite Compositions

**DOI:** 10.1021/acsaem.1c02375

**Published:** 2021-12-13

**Authors:** Ernestas Kasparavicius, Marius Franckevičius, Vida Malinauskiene, Kristijonas Genevičius, Vytautas Getautis, Tadas Malinauskas

**Affiliations:** † Department of Organic Chemistry, Kaunas University of Technology, Radvilenu pl. 19, Kaunas LT-50254, Lithuania; ‡ Department of Molecular Compound Physics, Centre for Physical Sciences and Technology, Saulėtekio Avenue 3, Vilnius LT-10257, Lithuania; § Institute of Chemical Physics, Faculty of Physics, Vilnius University, Sauletekio al. 3, Vilnius 10257, Lithuania

**Keywords:** perovskite solar cells, thermal stability, long-term stability, oxidized hole-transporting material, light absorption, conductivity

## Abstract

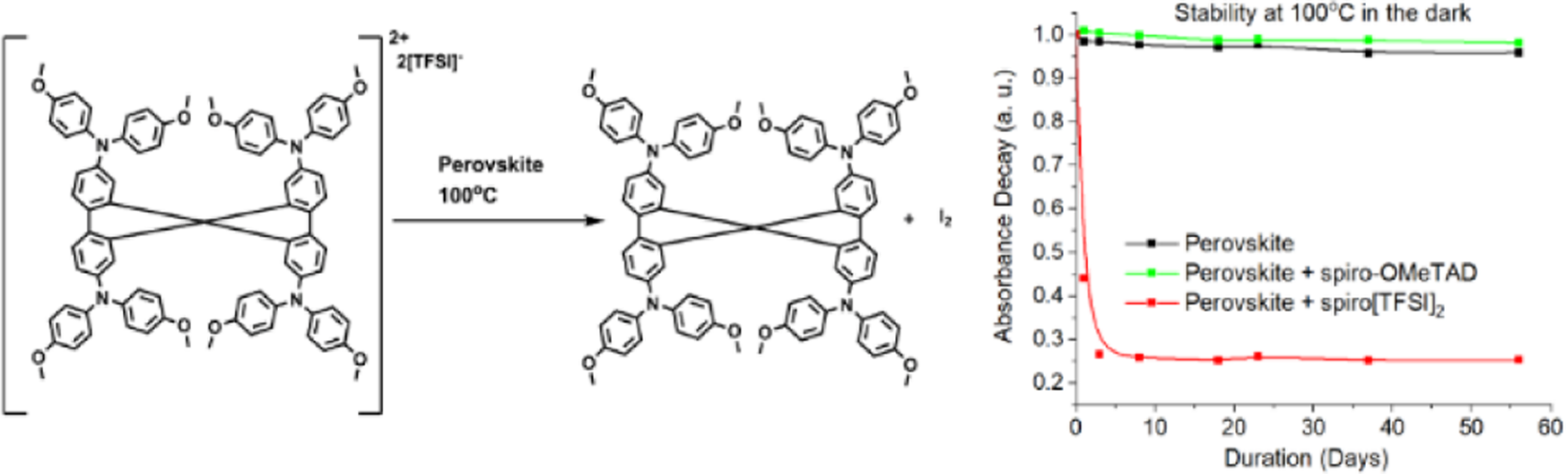

The power conversion efficiency of perovskite solar cells (PSCs) has risen steadily in recent years; however, one important aspect of the puzzle remains to be solved—the long-term stability of the devices. We believe that understanding the underlying reasons for the observed instability and finding means to circumvent it is crucial for the future of this technology. Not only the perovskite itself but also other device components are susceptible to thermal degradation, including the materials comprising the hole-transporting layer. In particular, the performance-enhancing oxidized hole-transporting materials have attracted our attention as a potential weak component in the system. Therefore, we performed a series of experiments with oxidized spiro-OMeTAD to determine the stability of the material interfaced with five most popular perovskite compositions under thermal stress. It was found that oxidized spiro-OMeTAD is readily reduced to the neutral molecule upon interaction with all five perovskite compositions. Diffusion of iodide ions from the perovskite layer is the main cause for the reduction reaction which is greatly enhanced at elevated temperatures. The observed sensitivity of the oxidized spiro-OMeTAD to ion diffusion, especially at elevated temperatures, causes a decrease in the conductivity observed in the doped films of spiro-OMeTAD, and it also contributes significantly to a drop in the performance of PSCs operated under prolonged thermal stress.

## Introduction

Perovskite solar cells (PSCs) have shown an impressive increase in device efficiency from 3.8 to 25.5% in recent years.^1^ The observed improvement is due to a combination of advantageous properties, such as long diffusion lengths for electrons and holes, high absorption coefficient, low material cost,^2−4^ and relative simplicity in the device fabrication.^5^ Although the efficiency of PSCs has steadily increased over the last decade, there is a significant interest in commercializing the technology. Unfortunately, the long-term stability of the devices is insufficient and it should be solved before market entry. A number of factors have been found to influence the extent of degradation and its rate, including moisture and oxygen ingress,^6^ UV light,^6^ electrical bias,^7−10^ temperature,^11,12^ and their variation.^13^ Obviously, understanding the underlying reasons for the observed instability and finding means to circumvent them is crucial for the future development of the PSC technology.

Temperature-induced degradation of PSCs is a particularly interesting and important topic, as temperatures as high as 40–45 °C can routinely occur under ambient conditions, while peak temperatures can be even higher, especially in hot desert environments, which are particularly attractive for photovoltaic energy generation due to the large number of sunshine hours and large unused area. Typically, photovoltaic devices are operated in direct sunlight, and the temperature of the panel can be up to 45 °C higher than the ambient temperature, exceeding 90 °C.^14^

Methylammonium lead iodide (MAPI) decomposes rapidly at temperatures around 135–150 °C, and even at 65–85 °C, slow decomposition is observed.^15,16^ Moreover, perovskites react with almost all metals, especially at moderate temperatures and illumination, as heat volatilizes the halide species and light increases the halogen mobility.^17−19^ Fortunately, the problems of thermal stability of perovskites could be alleviated and resistance to decomposition at operating temperatures improved by substituting methylammonium with formamidinium, Cs, or Rb.^20−22^

Unfortunately, not only the perovskite itself but also other device components are susceptible to thermal degradation, including the materials comprising the hole-transporting layer. Most commonly, hole-transporting materials (HTMs) are used with performance enhancing additives and dopants.^23^ At elevated temperatures, additives such as 4-*tert*-butylpyridine can evaporate, leading to the formation of voids^24,25^ or induce undesirable crystallization of the HTM in the film.^26^ Oxidized HTMs can react with 4-*tert*-butylpyridine to form pyridinated products.^27,28^ Moreover, lithium bis(trifluoromethanesulfonyl)imide (LiTFSI), another commonly used dopant, is quite hygroscopic, which further exacerbates the problem of moisture sensitivity of the perovskite.^29^

Diffusion of the mobile ions into the charge-transporting layers (CTLs) is another potential problem that is magnified at elevated temperatures.^30^ It has been shown that the migration of these ions can negatively affect the long-term stability of the PSC devices.^31−35^ It has been also reported that halides such as iodine and bromine are among the main culprits in elemental diffusion. The migration of these mobile ions beyond the perovskite/CTL interfaces can lead to an undesirable reaction between mobile ions and the CTL materials or metal electrode.^34,36,37^

Currently, 2,2′,7,7′-tetrakis(*N*,*N*-di-*p*-methoxyphenylamine)-9,9′-spirobifluorene (spiro-OMeTAD) is one of the most popular HTMs used in PSC research.^23^ To achieve the high efficiency devices, the conductivity of spiro-OMeTAD must be improved which is usually achieved by oxidizing a portion of the spiro-OMeTAD molecules using various dopants. Recently, it was observed that oxidized spiro-OMeTAD readily reacts with I- ions, migrating from MAPI under device operation, leading to a reduction of oxidized spiro-OMeTAD and a decrease in the conductivity of the transport layer.^32^ These diffusion and spiro-OMeTAD dedoping processes were observed to be thermally accelerated in MAPbI_3_ (MAPI) and FAPbI_3_ at elevated temperatures.^32,36,38^

It is evident that thermally accelerated ion diffusion processes observed in MAPI and FAPbI_3_ perovskites can lead to a significant long-term decrease in device performance due to the reduction of the oxidized spiro-OMeTAD. However, in recent years, a number of improved and more degradation-resistant perovskite compositions have been developed, particularly those containing cesium. Ion migration into the hole-transporting spiro-OMeTAD layer under thermal stress has not been studied in these new perovskites. Therefore, it is important to understand if the same issues exist in Cs-based perovskite systems. In the current publication, we investigate thermal stability of the oxidized spiro-OMeTAD in contact with various most popular perovskite compositions. Due to the complex nature of PSC devices and the multitude of possible processes occurring simultaneously, we have chosen to focus mostly on a specific problem of spiro-OMeTAD dedoping during the interaction between perovskite and oxidized spiro-OMeTAD in thin films at elevated temperatures, rather than attempting to evaluate the entire system at once.

## Results and Discussion

**Figure 1:**
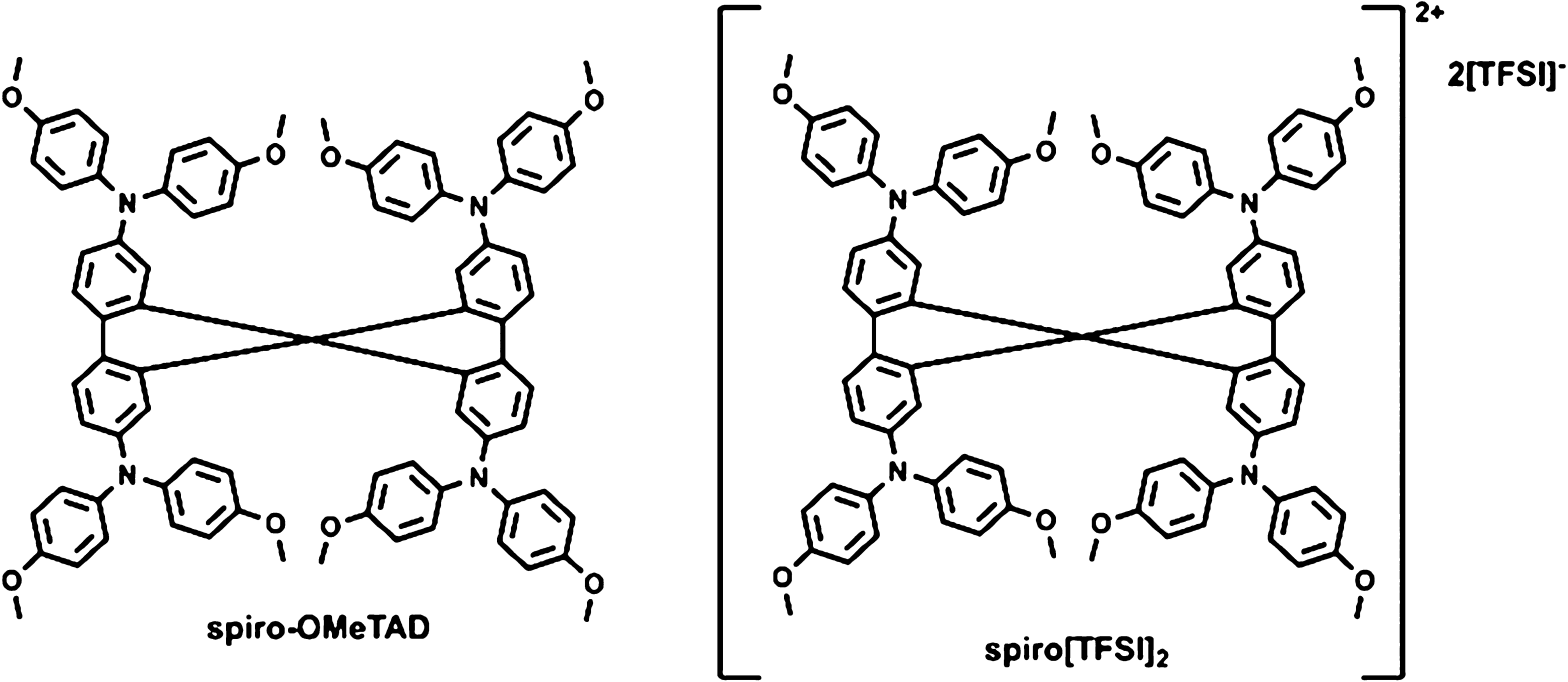
Structure of the investigated HTM spiro-OMeTAD and its oxidized analogue spiro[TFSI]_2_.

As mentioned in the Introduction, the interaction of oxidized spiro-OMeTAD with iodide ions migrating from MAPI at elevated temperatures leads to dedoping of the HTM, which is a major problem for the long-term stability of MAPI based PSCs. However, in recent years, a number of new perovskite compositions have been developed that exhibit improved stability compared to the original MAPI.^21,39^ Therefore, we decided to perform a series of experiments under different conditions to investigate the long-term stability of the oxidized HTM when interfaced with these new perovskites. Usually, oxidized HTM constitutes only a small part of the hole-transporting layer, the rest being regular neutral molecules. Therefore, it is quite difficult to monitor the degradation dynamics of oxidized HTM in the film in real time because their concentration is low and oxidized molecules are difficult to identify by standard analytical methods. For example, nuclear magnetic resonance (NMR) is not suitable at all due to the paramagnetic nature of the oxidized species, while identifying the oxidized species using mass spectrometry (MS) is difficult, as oxidized spiro-OMeTAD (spiro[TFSI]_2_) will only show the parent cation mass in MS and will start to revert back to spiro-OMeTAD in the HPLC column.^28,40,41^ Fortunately, cation radicals intensely absorb light in the visible region of the spectrum, and UV–vis spectroscopy could be effectively used to monitor changes in encapsulated systems without breaking encapsulation or degrading the samples. In our recent study, we investigated the stability of different oxidized HTMs and their behavior was very similar.^28^ Therefore, for this study, we focused on the most popular and intensively used spiro-OMeTAD as HTM. The oxidized spiro-OMeTAD (Figure 1), used in this study was synthesized by chemical oxidation with silver bis(trifluoromethanesulfonyl)imide.^40,41^

We first investigate the interaction of spiro[TFSI]_2_ with organic counterparts of perovskite such as methylammonium iodide (MAI), formamidinium iodide (FAI), and methylammonium bromide (MABr) by preparing solutions in acetonitrile. The initial experiments showed that the addition of FAI to the dissolved spiro[TFSI]_2_ caused an immediate color change (Figure S6a) as well as disappearance of the absorption maxima associated with oxidized HTM at about 510 nm and an enhancement of the absorption maxima associated with neutral spiro-OMeTAD at 380 nm (Figure S6b). A similar result was observed for MAI (Figure S7).

Processes that take place in solution do not necessarily take place on the surface of the solid film because the molecules are much more mobile and the interaction is easier and faster in the solution. Therefore, we additionally studied the interaction between the spiro[TFSI]_2_ film and solutions of FAI or MAI. To exclude the possibility of dissolution of the film by the solvent, we specifically chose a mixture of ethanol and H_2_O (1:1) to dissolve the organic salts but not the film of spiro[TFSI]_2_ (Figure S9). In both cases, spinning solution of the organic salts (MAI or FAI) on top of the spiro[TFSI]_2_ film resulted in immediate discoloration of the sample and disappearance of the characteristic absorbance maximum (Figures S10 and S11), indicating an interaction between spiro[TFSI]_2_ in the film and perovskite precursors. Similar experiments with solvent without MAI or FAI did not produce such results (Figure S9).

Interestingly, the substitution of iodine by bromine in MABr resulted in a significantly reduced reaction rate (Figures S8 and S12). The process, which took only minutes in the case of iodide-containing organic salts, lasted days to complete when MABr was used. Although a significant decrease in light absorption intensity at 510 nm is observed after the sample is kept at 100 °C for 24 h (under inert conditions), this indicates that the reaction is indeed taking place, albeit at a much slower rate, and that it is accelerated by increasing the temperature.

**Figure 2:**
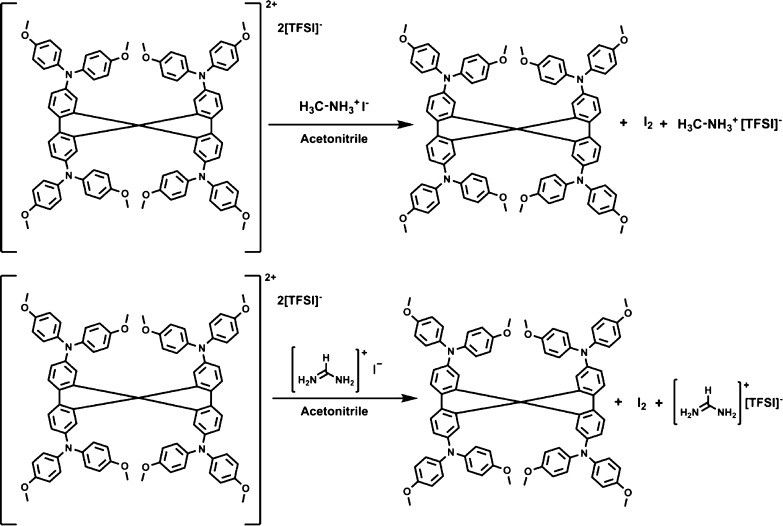
Proposed reaction of the oxidized spiro-OMeTAD with MAI and FAI.

To study the process in more detail and establish the final products, reactions with larger quantities of spiro[TFSI]_2_, MAI, and FAI were performed. After the reaction, one main product was isolated in very high yields. NMR and MS analyses (Figures S1–S3) identified that the reduction of spiro[TFSI]_2_ back to spiro-OMeTAD takes place during the interaction between spiro[TFSI]_2_ and MAI or FAI. A simple iodine test (Figure S5) and iodometric titration (see the Supporting Information for more details) indicated the formation of iodine during the process (Figure 2). We also performed MS analysis of the products obtained from the reaction between spiro[TFSI]_2_ and MAI, FAI, or MABr in solution. The interaction of spiro[TFSI]_2_ with MAI or FAI resulted only in the formation of the reduction product, namely, spiro-OMeTAD (Figures S33 and S34). Whereas during the slower reaction of spiro[TFSI]_2_ with MABr, both spiro[TFSI]_2_ and spiro-OMeTAD were detected (Figures S31 and S32). Overall, experiments with the organic halide precursors demonstrate what sort of processes occur and do they take place in the solid state where molecules are a lot less mobile.

It is interesting that oxidized HTM reacts so readily with perovskite precursors; however, the more important question is whether the same process occurs in the solid state between the complete perovskite and spiro[TFSI]_2_ films, as this is more relevant to the actual PSC devices. For this reason, we have performed experiments in the solid state with oxidized spiro-OMeTAD and five different popular perovskite compositions: Cs_5_(MA_0.17_FA_0.83_)_95_Pb(I_0.83_Br_0.17_)_3_ (CsMAFA), MA_0.17_FA_0.83_Pb(I_0.83_Br_0.17_)_3_ (MAFA), FA_0.83_Cs_0.17_Pb(I_0.83_Br_0.17_)_3_ (CsFA), MAPI, and MAPbBr_3_ (MAPB).

Due to the complex nature of PSC devices, multiple processes take place simultaneously during device operation; therefore, we first chose to focus specifically on the stability of oxidized spiro-OMeTAD in contact with the perovskite film, rather than trying to evaluate the undergoing processes in the complete device. If observed reactions do take place in the described setup, it could be a very strong indicator that the same processes will happen in the PSCs, although during a longer time frame, as concentration of the oxidized spiro-OMeTAD in the actual devices is lower.

Investigated spiro[TFSI]_2_ has been deposited as a thin film on top of the perovskite. The glass substrate with two deposited layers was encapsulated under an inert atmosphere with a second glass substrate and stored under different conditions for almost 2 months (see the Supporting Information for more details). The UV–vis spectra of the studied samples were recorded periodically during the experiments (Figures S13–S27), and the results were compared with two different reference samples: the pure perovskite film and spiro-OMeTAD film on top of the perovskite. The absorption of the neutral and oxidized spiro-OMeTAD compounds was measured at the corresponding absorption maxima of 380 and 510 nm, respectively. The perovskite absorption was followed at 590 nm so that it would not overlap with the absorption maxima of spiro-OMeTAD and spiro[TFSI]_2_; the only exception is MAPbBr_3_, which was followed at 510 nm due to its narrower absorption interval. Thinner perovskite films were produced (see the Supporting Information for more details) so that light absorbance by the perovskite would not interfere too significantly with that of spiro-OMeTAD and spiro[TFSI]_2_.

**Figure 3:**
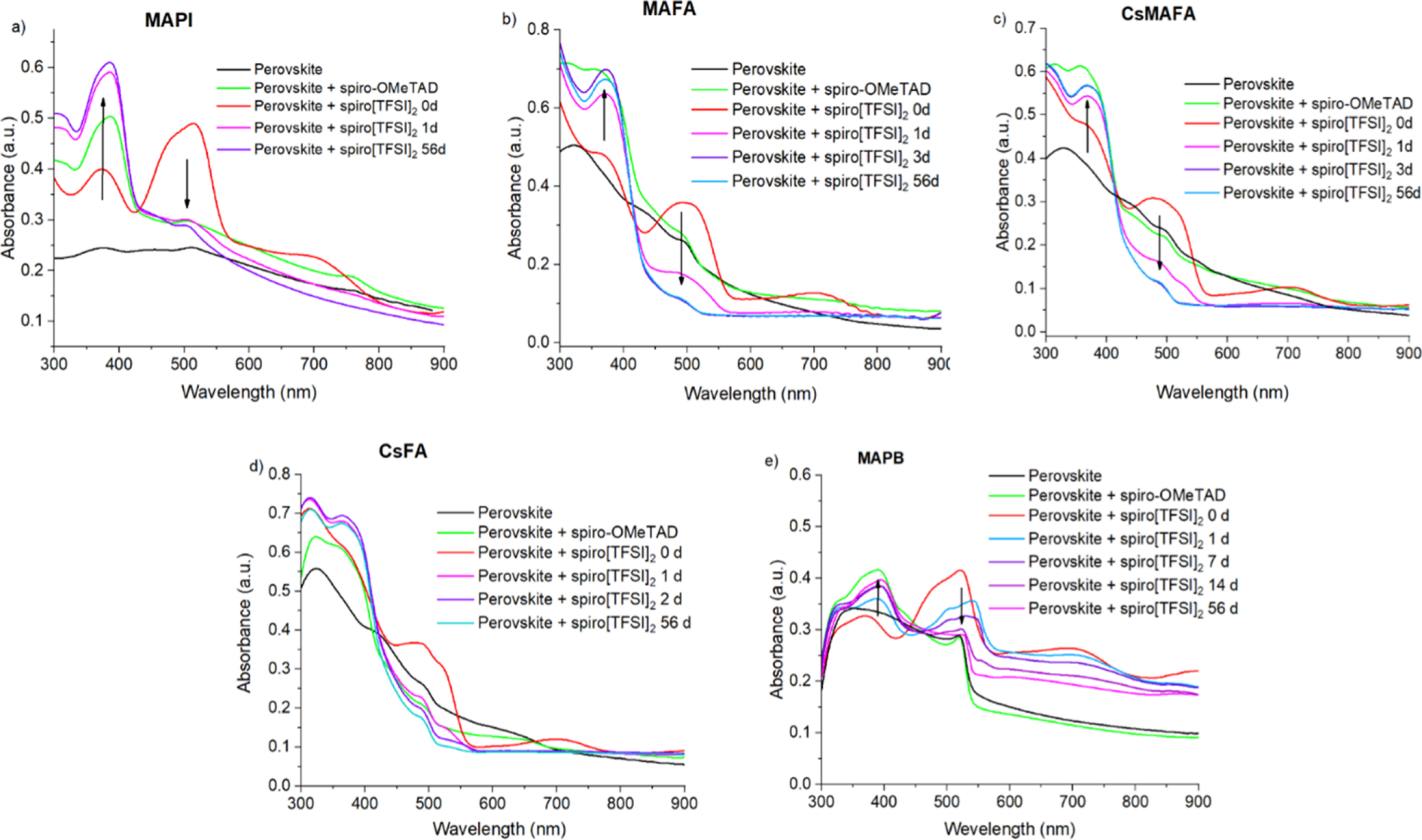
Change in light absorption of the encapsulated spiro[TFSI]_2_ films on various perovskites, MAPI (a), MAFA (b), CsMAFA (c), CsFA (d), and MAPB (e), at 100 °C in the dark.

**Figure 4:**
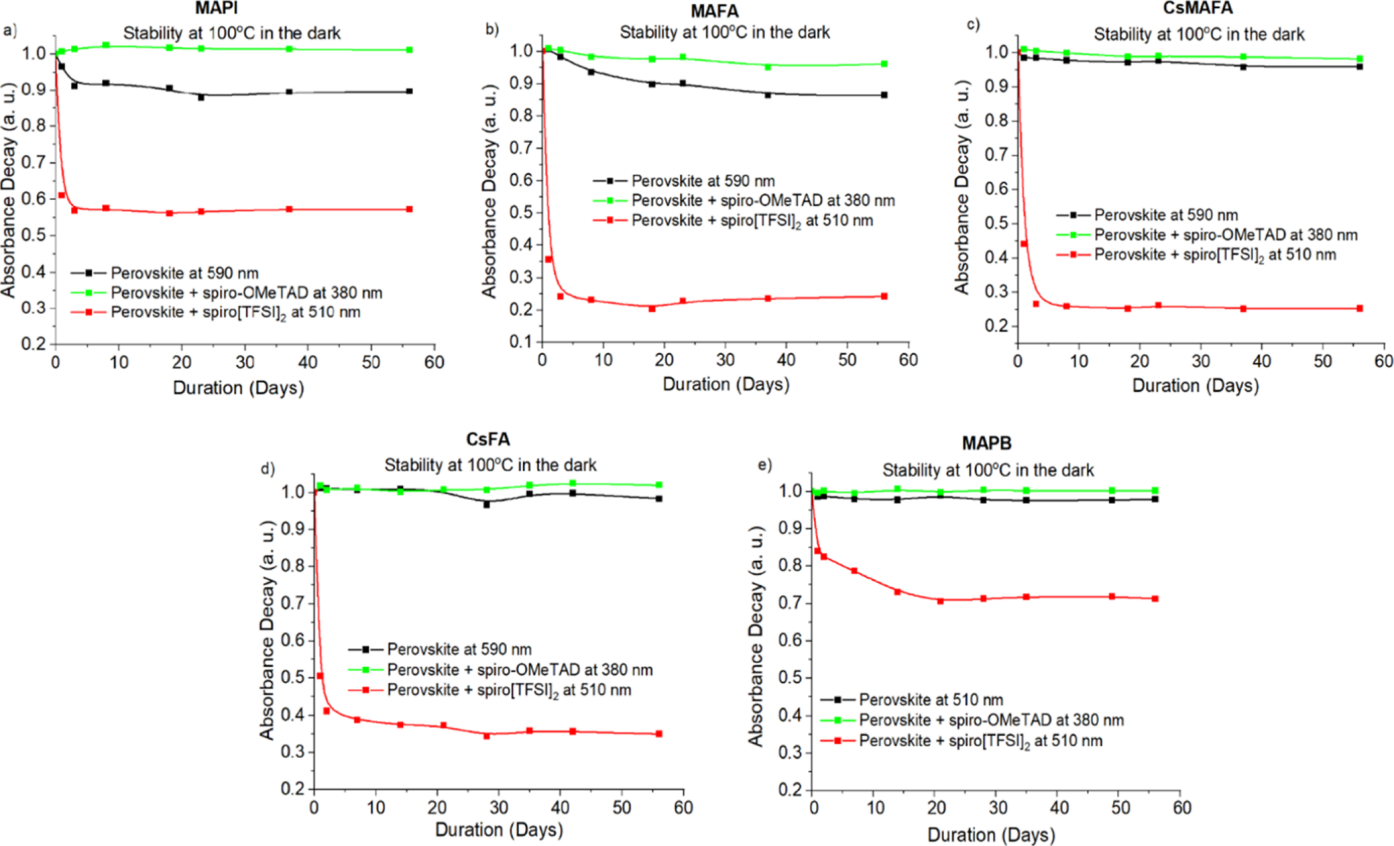
Light absorption intensity dynamics at 100 °C temperature in the dark of the spiro[TFSI]_2_ or spiro-OMeTAD films on different perovskites: MAPI (a), MAFA (b), CsMAFA (c), CsFA (d), and MAPB (e).

**Figure 5:**
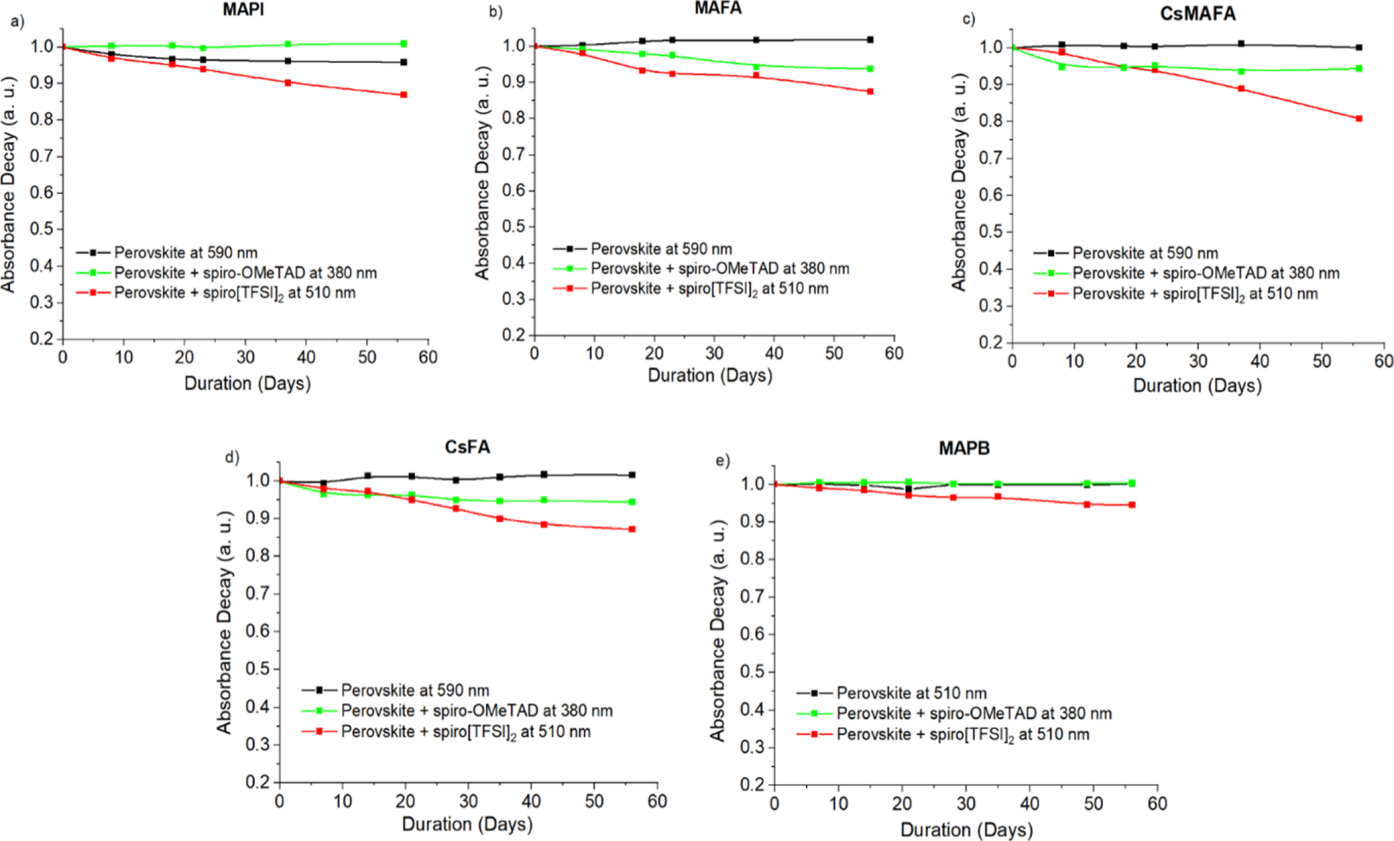
Light absorption intensity dynamics of spiro[TFSI]_2_ and spiro-OMeTAD layers on different perovskites, MAPI (a), MAFA (b), CsMAFA (c), CsFA (d), and MAPB (e), obtained at room temperature under daylight conditions.

Experiments with spiro[TFSI]_2_ on MAPI at 100 °C in the dark showed that there is a rapid decrease in absorption intensity at 510 nm and an increase at 380 nm (Figures 3a and 4a), indicating the reduction of the oxidized species and the formation of the neutral spiro-OMeTAD. The whole process lasted less than 1 day, and only minor changes in the UV–vis spectrum were observed afterward. Similar processes, although not as intense and fast, can be observed in samples kept under illumination at room temperature (RT) (Figure 5a) and even in the dark (Figure S28a)

The migration of iodide ions in the solid state at elevated temperature is sufficiently intense to reduce spiro[TFSI]_2_ to the neutral molecule in a few hours. Even at RT, the process can be observed for extended periods of time. Similar to reports in the literature,^15,16^ a decrease in perovskite absorption intensity is also observed at elevated temperatures (Figures 4a and S13c), indicating thermal stability related issues with MAPbI_3_.

A similar picture can be observed using FA- and MA-containing perovskite MAFA (Figures 3b and 4b), where the absorption intensity of spiro[TFSI]_2_ decreases more rapidly under thermal stress rather than under direct sunlight at RT (Figure 5b). Similarly to MAPbI_3_, MA- and FA-containing perovskite also demonstrates some loss of absorption intensity at elevated temperatures, again indicating sensitivity of the material to elevated temperatures over extended periods of time (Figures 4b and S16c).

The introduction of the Cs cation into the CsMAFA and CsFA perovskite compositions results in improved stability of the materials under prolonged thermal stress (Figure 4c,d). However, iodide ions still diffuse into the adjacent spiro[TFSI]_2_ layer, promoting relatively rapid reduction of the material at 100 °C (Figures 3c,d and 4c,d). Similarly as with other types of perovskites, ion migration undergoing at RT deteriorates spiro[TFSI]_2,_ which is confirmed by the decrease in the intensity of the absorption peak at 510 nm (Figures 5c,d and S29).

Interestingly, the perovskite films with spiro-OMeTAD show very little change during prolonged tests (Figures 4 and 5), suggesting that the neutral molecule is sufficiently stable under the conditions studied.

During these experiments, we did not observe any significant crystallization of the oxidized spiro-OMeTAD, as it takes about a week for it to become noticeable,^28^ and in the current investigation, we saw that oxidized spiro-OMeTAD is reduced back to spiro-OMeTAD much faster (in 1–2 days). Additionally we did not use 4-*tert*-butylpyridine (tBP) which accelerates the crystallization of both spiro[TFSI]_2_ and spiro-OMeTAD.^26^

The substitution of the iodide with bromide ion in the MAPB perovskite results in improved resistance of the perovskite material to thermal stress (Figure 4e). The lower reactivity of the bromide ensures that the reduction of spiro[TFSI]_2_ at 100 °C is much slower and takes days rather than hours (Figures 3e and 4e), while only a slight change in absorption intensity is observed at RT (Figure 5e).

To confirm that the same reaction occurs in the solid films as in the solution, HPLC-MS analysis of the samples was performed after stability experiments. The identification of the oxidized material is problematic because spiro[TFSI]_2_ starts to change back to a neutral molecule in the HPLC column and only the mass of the parent molecule can be observed in MS.^40,41^ Fortunately, the reduction process in the column is slow enough to detect two elution peaks at different retention times one for spiro[TFSI]_2_ (retention time 6.9 min) and another for spiro-OMeTAD (retention time 7.6 min) (Figures S40 and S41), both giving 1225 mass spiro-OMeTAD molecular cation [M + H]^+^.

Only neutral spiro-OMeTAD was detected in all of the samples with spiro[TFSI]_2_ on different perovskite compositions stored at 100 °C for 56 days (Figures S35–S39). The results of HPLC-MS analysis correlate well with the data obtained from UV–vis spectroscopy.

**Figure 6:**
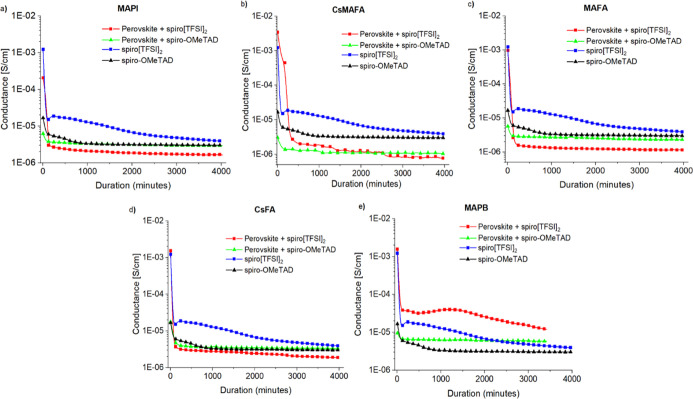
Change in conductivity of the spiro[TFSI]_2_ or spiro-OMeTAD encapsulated films on different perovskites, MAPI (a), MAFA (b), CsMAFA (c), CsFA (d), and MAPB (e), at 100 °C in the dark.

In addition to UV–vis measurements, the changes in conductivity were also investigated. Oxidized and pristine spiro-OMeTAD were deposited on top of the different perovskite compositions and conductivity was measured at an elevated temperature (100 °C) in the dark (Figure 6). In addition, samples containing only pristine or oxidized HTM on glass substrate were also measured.

The observed conductivity of the samples, containing the perovskite compositions MAFA, CsFA, and MAPI and a layer of spiro[TFSI]_2_, degrade very rapidly. During the first hour, it decreases by several orders of magnitude and becomes very similar to that registered for spiro-OMeTAD (Figure 6a,c,d). Interestingly, spiro[TFSI]_2_ with the perovskite composition CsMAFA performs slightly better (Figure 6b), and its decay is somewhat slower, although the final result is very similar to those previously discussed. As expected from the UV–vis data, spiro[TFSI]_2_ with MAPB shows the slowest decrease in conductivity (Figure 6e). It is worth noting that the conductivity of the pristine spiro[TFSI]_2_ also diminishes with time, although not as rapidly as in the samples with perovskite absorber. Overall, due to increased temperature,^42^ somewhat lower conductivity is measured at 100 °C, compared with the results recorded at room temperature (Table S1).

We have additionally performed stability tests on the complete solar cell devices based on the triple cation CsMAFA and the classical MAPI compositions. The PSCs of each composition were constructed using three different HTM variations: undoped spiro-OMeTAD, spiro[TFSI]_2_, or spiro-OMeTAD doped with typical dopants used in PCS research, tBP, LiTFSI, and tris[2-(1*H*-pyrazol-1-yl)-4-*tert*-butylpyridine]cobalt(III) (FK-209). Two different environmental conditions were used to study the long-term stability of the devices: (i) the devices were stored in air under light at 90 °C and (ii) the devices were stored in nitrogen at 90 °C without light. We investigated the stability of the solar cells by monitoring the power conversion efficiency over time. Figure S45 shows the photovoltaic stability data of the different PSCs obtained for each condition. The current–voltage characteristics of the devices are shown in Figures S43 and S44, while the photovoltaic parameters are summarized in Tables S2–S13.

The solar cells kept under ambient conditions were particularly unstable (Figure S45a,b). Both the MAPI and CsMAFA devices experienced significant efficiency degradation over time and after 50 h of continuous aging, their efficiency has halved. To see the influence of the temperature alone, we also kept the devices under the elevated temperature while suppressing other environmental factors. It is evident that the devices that were exclusively exposed to elevated temperatures (Figure S45c,d) show improved stability compared to the ones kept under ambient conditions, which is mainly caused by the reduced photooxidation-induced degradation of the perovskite. If we focus on the HTM present in the devices, as shown in Figure S45c,d, the degradation of both MAPI and CsMAFA PSCs containing doped spiro-OMeTAD is faster than those with undoped spiro-OMeTAD, especially at longer times. The poor stability is most likely related to the presence of oxidized spiro-OMeTAD as well as dopants (such as LiTFSI and tBP), which can be released over time, degrading the interfaces and reducing device performance. The stability of oxidized spiro-OMeTAD is worse than the undoped analogue, as it is reduced back to the neutral molecules as discussed above. However, its stability tends to be better than doped spiro-OMeTAD, suggesting that the reduction of not only the oxidized species but also dopants has significant impact on device stability at elevated temperatures.

As the presence of oxidized HTM molecules in the CTL significantly improves conductivity,^40,43^ the observed sensitivity of oxidized spiro-OMeTAD to ion diffusion from the perovskite, especially under thermal stress, is among the main causes of conductivity decrease in the doped films of spiro-OMeTAD.^38^ Additionally, it is one of the important reasons for decline in performance of PSCs operated under prolonged thermal stress.

In the broader context of research performed recently addressing stability issues, at the first glance, results reported in this publication would seem to contradict them. However, if we look at the device stability curves presented in the research articles describing PSCs containing doped spiro-OMeTAD, for example, Christians et al.,^44^ Seo et al.,^45^ and Kong et al.,^46^ there is a slow but consistent decrease in device performance. This drop in performance can be attributed to more than one factor; however, it does not eliminate the prospect that processes described in this publication are also playing a role. We also have to consider the fact that, aging conditions used in this publication were harsher, which in turn led to faster degradation of the devices. Furthermore, concentration of oxidized HTM is significantly lower (10% or less^40^) in the devices constructed using doped spiro-OMeTAD, so processes described in this publication would take substantially longer to manifest in full.

A possible solution could be the use of charge-transporting materials that do not rely on doping and oxidized molecules to function effectively in PSC.^47^ Alternatively, suppression of ion migration via low-dimensional diffusion barriers,^48^ blocking layer,^6,49^ or defect passivation^50,51^ could be another option. Finally, the use of charge-transporting materials capable of forming a self-assembled monolayer could simultaneously solve both doping and HTM film quality issues.^52,53^

## Conclusions

In conclusion, a series of experiments were performed with oxidized spiro-OMeTAD to determine the stability of the material in contact with various most popular perovskite compositions. Particular attention was given to the long-term stability of the material under thermal stress. It was found that oxidized spiro-OMeTAD is readily reduced to the neutral molecule upon interaction with the perovskite precursors MAI and FAI in the solution as well as with all five perovskite compositions in the solid state. We have found that the presence of iodide ions, either in solution or by ion migration from the perovskite layer, is the main cause of the dedoping process. During the reaction, oxidized spiro-OMeTAD is reduced back to the neutral molecule and iodine is formed. In the thin films, ion diffusion and consequently reduction of oxidized spiro-OMeTAD is greatly enhanced at elevated temperatures, leading to a complete disappearance of spiro[TFSI]_2_ within a few days. The observed processes take place for all perovskite compositions, although it is worth mentioning that spiro[TFSI]_2_ degrades significantly slower in contact with MAPB due to the lower reaction rate with the bromide ion. Because the presence of oxidized HTM molecules in the CTL significantly improves the conductivity, the observed sensitivity of spiro[TFSI]_2_ to ion diffusion from the perovskite, especially under thermal stress, could be one of the reasons for the decrease in conductivity observed in the doped films of spiro-OMeTAD and the subsequent decrease in the performance of PSCs operated at elevated temperatures. Possible solutions to this problem could be the use of materials that do not require doping and therefore do not rely on oxidized materials to improve conductivity or the suppression of ion diffusion between layers.

## Supporting Information Available

Experimental section, synthetic procedures, NMR spectra, mass spectra, information on preparation of HTM and perovskite films, HTM solutions, qualitative and quantitative determination of iodine, preparation of PSCs, absorption spectra in solution and solid state, and solar cell characteristics (PDF)
