# Supplementary material for: Oxidized Spiro-OMeTAD: Investigation of Stability in Contact with Various Perovskite Compositions
Source: ACS Appl Energy Mater. 2021 Dec 13;4(12):13696–705. doi: 10.1021/acsaem.1c02375 (PMC8715445; doi:10.1021/acsaem.1c02375)
Supplement: Supplementary file 1 — ae1c02375_si_001.pdf [file ae1c02375_si_001.pdf]

Supporting information

Oxidized spiro-OMeTAD, Investigation of Stability in Contact with Various Perovskite Compositions

Ernestas Kasparavicius[a], Marius Franckevičius[b], Vida Malinauskiene[a], Kristijonas Genevičius[c], Vytautas Getautis[a], Tadas Malinauskas[a] *

*Email: tadas.malinauskas@ktu.lt

[a]Department of Organic Chemistry, Kaunas University of Technology, Radvilenu pl. 19, Kaunas, LT-50254, Lithuania

[b]Department of Molecular Compound Physics, Centre for Physical Sciences and Technology, Saulėtekio Avenue 3, Vilnius LT-10257, Lithuania

[c]Institute of Chemical Physics, Faculty of Physics, Vilnius University Sauletekio al. 3, Vilnius 10257, Lithuania

General methods and materials

All reagents were purchased from commercial companies. The 1H and 13C NMR spectra were taken on Bruker Avance III 400 instrument (400 MHz for 1H, 100 MHz for 13C) spectrometer at RT. All the data are given as chemical shifts in δ (ppm). The course of the reactions was monitored by TLC on ALUGRAM SIL G/UV254 plates and developed with UV light. Silica gel (grade 9385, 230–400 mesh, 60 Å, Aldrich) was used for column chromatography. Elemental analysis was performed with an Exeter Analytical CE-440 elemental analyser, Model 440 C/H/N/. Thermogravimetric analysis (TGA) was performed on a Q50 thermogravimetric analyzer (TA Instruments) at a scan rate of 10 K min^{-1} in the nitrogen atmosphere. UV-Vis spectra were recorded on a Shimadzu UV-2600 spectrophotometer. Microcells with an internal width of 1 mm were used. Optical microscopy experiments were conducted on Olympus BX-41 equipped with QImaging Go-3 camera. Thickness of the films was measured using MarSurf WS1 white light interferometer. MS spectroscopy was done using Waters HPLC-system with UV (200-700 nm) and MS (20-2000 m/z) detectors. MS investigation of the films was done after stability experiments lasting 56 days. The samples have been prepared by breaking open encapsulation and dissolving the films in acetonitrile. Conductivity measurements of the hole transporting layers were performed by the two-probe method using Keithley 2602A source meter. The distance between the golden electrodes in all structures was 1mm, the length of the contacts was around 10mm. The samples were placed on a hot stage, temperature was measured by a thermocouple and controlled by a PID controller. Two encapsulated samples with oxidized and non-oxidized spiro-OMeTAD on the same perovskite layer were measured simultaneously, three examples of each were studied. The degradation was performed at 100°C for about 4000

minutes and conductivity was measured in dark without switching off the temperature and without taking samples from hot stage.

The oxidized derivative of spiro-OMeTAD used in the study (spiro[TFSI]$_2$), has been obtained using chemical oxidation by the silver bis(trifluoromethanesulfonyl)imide (AgTFSI) [1].

The organic cations were purchased from Dyesol; the lead compounds from TCI; CsI from abcr GmbH.

Substrate preparation for the long-term stability testing

Corning Glass 0215 substrates were cleaned by sonication in 2% Hellmanex water solution for 30°min. After rinsing with deionized water and ethanol, the substrates were further cleaned by sintering at 500°C for 30 min and left to cool down to room temperature.

Preparation of perovskite films containing caesium $Cs_{0.05}(MA_{0.17}FA_{0.83})_{0.95}Pb(I_{0.83}Br_{0.17})_3$ (CsMAFA) and analogue without caesium $MA_{0.17}FA_{0.83}Pb(I_{0.83}Br_{0.17})_3$ (MAFA)

The perovskite precursor films were deposited from a precursor solution containing FAI 0.017g (0.1 M), PbI_2 0.051 (0.11 M), MABr 0.002g (0.02 M) and $PbBr_2$ 0.007g (0.02 M) in anhydrous DMF:DMSO (4:1 volume ratio) (1ml). According to the article, this composition contains a lead excess as reported elsewhere [2, 3]. Then CsI, predissolved as a 0.15 M stock solution in DMSO, was added to the mixed perovskite precursor to achieve the desired triple cation composition [4], in the samples without Cs this step was omitted.

The perovskite solution was spin coated in a two-step program at 1000 and 6000 rpm for 10 and 25 s respectively. During the second step, 200 µL of chlorobenzene was poured on the spinning substrate 5 s prior to the end of the program. Films with Cs-containing perovskite turned dark immediately after spin coating. The substrates were then annealed at 100 °C for 1 h in a argon atmosphere.

Preparation of $MAPbI_3$ (MAPI) perovskite films

The MAPI perovskite precursor solutions were prepared by solubilizing the mixture of PbI_2 0.092 g (0.1 M) and CH_3NH_3I 0.032 (0.1 M) in DMF 2 ml. The perovskite solution was spin coated in a two-step program at 1000 and 6000 rpm for 10 and 25 s respectively. The substrates were then annealed at 100 °C for 1 h.

Preparation of $FA_{0.83}Cs_{0.17}Pb(I_{0.83}Br_{0.17})_3$ (CsFA)perovskite films

CsFA perovskite was made according to the publication [5]. A solution of precursor was prepared with caesium iodide 0.013g (0,025M), lead bromide 0.021 g (0,028 M), formamadinium iodide 0.041 g (0,12M), and lead iodide 0.108 g (0,117M). Precursors were dissolved in solution (2ml) of DMF and DMSO (4:1 volume ratio) by stirring at 70 °C for 15 minutes. Precursor solution was spread onto substrates and spin coated at 1000 rpm for 10 s (1000 rpm s^{-1} ramp) followed immediately by 6000 rpm for 35 s. 10 seconds before the end of spin coating, the films were solvent quenched with 100 µl of anhydrous anisole. Substrates were annealed at 100 °C for 15 minutes. All spin coating occurred at 30-40% relative humidity.

Preparation of $MAPbBr_3$ (MAPB)perovskite films

MAPB film was deposited using 1-step deposition method. 0,367g (0.5 M) $PbBr_2$ precursor solution was prepared in 2ml of DMF and DMSO mixture (4:1 volume ratio) without stirring at 80°C for 20 minutes until completely dissolved. After cooling down at room temperature, this solution was mixed with 0,111g (0.5 M) of MABr powder to obtain a final solution. The perovskite solution was spin coated in a two-step program at 1000 and 4000 rpm for 10 and 40 s respectively. The substrates were then annealed at 100 °C for 3min to obtain shiny and homogenous orange film. [6]

HTM film preparation for stability testing with various perovskites

The investigated oxidized charge transporting material spiro[TFSI]$_2$ has been dissolved in acetonitrile (20 mg/ml) and immediately spin-coated onto layer formed from different perovskites. spiro-OMeTAD has been dissolved in chlorbenzene (14 mg/ml) and immediately spin-coated at 2000 rpm for 20 s onto layer formed from different perovskites.

Thickness of the films was measured to be ~50 nm. All prepared films have been encapsulated under inert atmosphere using a second glass substrate and UV curable epoxy (Ossila Ltd., UK) for edge sealing. Care has been taken to avoid contact between edge sealant and investigated material, as epoxy dissolves the HTM film. Three samples of each type were made and have been kept at RT in the dark and under ambient light as well as at 100°C in the dark for 56 days and UV-Vis absorption spectra were recorded periodically. MS samples of the investigated films, after 56 days, have been prepared by breaking open encapsulated sample and dissolving the films with acetonitrile.

Influence of individual perovskite components on stability of spiro[TFSI]$_2$ films

For the experiments involving interaction between films of oxidized charge transporting material spiro[TFSI]$_2$ and organic cations, the appropriate organic cation (FAI 0.042g, 0.5 M or MAI 0.039g, 0.5 M or MABr 0.028g, 0.5 M) dissolved in 0.5 ml of the mixture of H_2O/EtOH (1:1 volume ratio) and was deposited on top of HTM via spin coating at 2000 rpm for 20 s. Mixture of ethanol and water was used as a solvent as not to dissolve the layer of spiro[TFSI]$_2$ already deposited.

Film preparation for the conductivity measurements

Corning Glass 0215 substrates were cleaned by sonication in 2% Hellmanex water solution for 30ºmin. After rinsing with deionized water and ethanol, the substrates were further cleaned by sintering at 500°C for 30 min and left to cool down to room temperature.

Perovskite films containing caesium CsMAFA and analogue without caesium MAFA were prepared according to the literature [4]. The MAPI perovskite films were formed according to A. Abate et all [7]. CsFA perovskite was made according to the publication [5]. MAPB film was deposited using 1-step deposition method according to the publication[6].

The investigated oxidized charge transporting material spiro[TFSI]$_2$ has been dissolved in acetonitrile (20 mg/ml) and immediately spin-coated onto layer formed from different perovskites. spiro-OMeTAD has been dissolved in chlorbenzene (14 mg/ml) and immediately spin-coated at 2000 rpm for 20 s onto layer formed from different perovskites. Thickness of the

films was measured to be ~50 nm, a gold electrode (~20 nm) has been evaporated on top of charge transporting material.

All prepared films have been encapsulated under inert atmosphere using a second shorter glass substrate and UV curable epoxy (Ossila Ltd., UK) for edge sealing. Care has been taken to avoid contact between edge sealant and investigated material, as epoxy dissolves the HTM film. The samples have been kept at 100°C in the dark for 4000 minutes and conductivity measurements automatically was performed every 90 minutes.

Qualitative determination of I_2 in solution

6 mg of spiro[TFSI]$_2$ was dissolved in 1 ml of acetonitrile, 3 mg of FAI or MAI were added and the solution changed colour from dark red to yellow. 0.1 ml of the prepared sample were added to 1 ml of 4% starch solution colouring it in purple indicating that iodine was formed during the reaction.

Preparation of the solutions for the experiments of spiro[TFSI]$_2$ reduction with organic cations, monitored via UV-Vis absorption spectroscopy

100 ml of 10^{-4} M (0.01 mmol) spiro[TFSI]$_2$ solution in acetonitrile was divided into four 25 ml volumetric flasks. In order for the spiro[TFSI]$_2$ to react fully, two equivalents of MAI (0.0025 mmol*158.97 g/mol*2 = 0.794 mg), FAI (0.0025 mmol*171.97 g/mol*2=0.85985 mg) or MABr (0.0025 mmol*111.97 g/mol*2= 0.55985 mg) were calculated. Stock solution with 5-fold excess of appropriate halide was prepared in 1ml of acetonitrile and 0.2 ml of the solution was added to

the spiro[TFSI]$_2$ sample, UV-Vis spectra were recorded before and after the addition of the halide solution. When 0.2 ml of FAI solution was added to the spiro[TFSI]$_2$ sample, the colour of the sample faded. UV was recorded before and after the reaction.

Determination of iodine content in the mixtures of spiro[TFSI]$_2$ and FAI by iodometric titration

General procedure

0.01 N solution of $Na_2S_2O_3$ in water was prepared using $Na_2S_2O_3$ * $5H_2O$ fixanal. Sample of spiro[TFSI]$_2$ is dissolved in 1 ml of acetonitrile (dark red solution), 5 mg (0,029 mol) of FAI is added to the solution and it changes colour to yellow. The solution is diluted with 2 mL of H_2O and 5 drops of 4% starch solution are added, turning the starch suspension dark purple. The sample is titrated with the prepared sodium thiosulphate solution until the violet colour of the starch suspension disappears.

spiro[TFSI]$_2$ + 2FAI → spiro-OMeTAD + 2FA[TFSI]+ I_2

$I_2 + Na_2S_2O_3 = 2NaI + Na_2S_4O_6$

Experiment 1

Violet colour of the sample disappears after 0.71 ml of sodium thiosulphate solution was used for titration.

Amount of sodium thiosulphate in 250 ml of 0.01 N solution was:

m= 0.01N×124.09 g/eq×0.25l=0.310225 g

0.71 ml of sodium thiosulphate 0.01 N solution used in titration is:

m= 310.225mg×0.71ml/250ml=0.881 mg (0.00355 mmol)

According to the reaction equation 0.00355 mmol of spiro$[TFSI]_2$ or m=0.00355×1785.77g/mol= 6.34mg have reacted with FAI. We have used 6.45 mg of spiro$[TFSI]_2$, therefore X_1=6.34mg×100%/6.45mg=98.28% of the starting material have been used up in the reaction.

Experiment 2

Violet colour of the sample disappears after 0.7 ml of sodium thiosulphate solution was used for titration. Amount of sodium thiosulphate in 250 ml of 0.01 N solution was:

m= 0.01N×124.09 g/eq×0.25l=0.310225 g

0.7 ml of sodium thiosulphate 0.01 N solution used in titration is:

m= 310.225mg×0.7ml/250ml=0.87 mg (0.00349 mmol)

According to the reaction equation 0.00349 mmol of spiro$[TFSI]_2$ or m=0.00349×1785.77g/mol= 6.25mg have reacted with FAI. We have used 6.34 mg of spiro$[TFSI]_2$, therefore X_2=6.25mg×100%/6.34mg=98.58% of the starting material have been used up in the reaction.

Experiment 3

Violet colour of the sample disappears after 0.72 ml of sodium thiosulphate solution was used for titration. Amount of sodium thiosulphate in 250 ml of 0.01 N solution was:

m= 0.01N×124.09 g/eq×0.25l=0.310225 g

0.72 ml of sodium thiosulphate 0.01 N solution used in titration is:

m= 310.225mg×0.72ml/250ml=0.89 mg (0.0036 mmol)

According to the reaction equation 0.0036 mmol of spiro$[TFSI]_2$ or m=0.0036×1785.77g/mol= 6.42mg have reacted with FAI. We have used 6.52 mg of spiro$[TFSI]_2$, therefore X_3=6.42mg×100%/6.52mg=98.6% of the starting material have been used up in the reaction.

The experiment was repeated 3 times in total and X_1= 98.28 %; X_2 = 98.58%, X_3=98.6% gives $X_{average}$= 98,48 ±0,18%, indicating good correlation between amount of spiro[TFSI]$_2$ used and iodine produced during reaction as well as proving the validity of the proposed reaction that takes place between spiro[TFSI]$_2$ and FAI.

Determination of iodine content in the mixtures of spiro[TFSI]$_2$ and MAI by iodometric titration

General procedure

0.01 N solution of $Na_2S_2O_3$ in water was prepared using $Na_2S_2O_3$ * $5H_2O$ fixanal. Sample of spiro[TFSI]$_2$ is dissolved in 1 ml of acetonitrile (dark red solution), 5 mg (0,031 mol) of MAI are added to the solution and it changes colour to yellow. The solution is diluted with 2 mL of H_2O and 5 drops of 4% starch solution are added, turning the starch suspension dark purple. The sample is titrated with the prepared sodium thiosulphate solution until the violet color of the starch suspension disappears.

Spiro[TFSI]$_2$ + 2MAI→ Spiro-OMeTAD + 2MA[TFSI]+ I_2

$I_2 + Na_2S_2O_3 = 2NaI + Na_2S_4O_6$

Experiment 1

Violet colour of the sample disappears after 0.71 ml of sodium thiosulphate solution was used for titration.

Amount of sodium thiosulphate in 250 ml of 0.01 N solution was:

$m = 0.01N \times 124.09\ g/eq \times 0.25l = 0.310225\ g$

Amount of sodium thiosulphate in 0.71 ml of 0.01 N solution was:

$m = 310.225mg \times 0.71ml/250ml = 0.881\ mg$ (0.00355 mmol)

According to the reaction equation 0.00355 mmol of spiro[TFSI]$_2$ or $m=0.00355 \times 1785.77 g/mol=$ 6.34 mg have reacted with MAI. We have used 6.37 mg of spiro[TFSI]$_2$, therefore $X_1=6.34mg \times 100\%/6.37mg=99.52\%$ of the starting material have been used up in the reaction.

Experiment 2

Violet colour of the sample disappears after 0.73 ml of sodium thiosulphate solution was used for titration.

Amount of sodium thiosulphate in 250 ml of 0.01 N solution was:

$m = 0.01N \times 124.09\ g/eq \times 0.25l = 0.310225\ g$

Amount of sodium thiosulphate in 0.73 ml of 0.01 N solution was:

$m = 310.225mg \times 0.73ml/250ml = 0.90\ mg$ (0.00365 mmol)

According to the reaction equation 0.00365 mmol of spiro[TFSI]$_2$ or $m=0.00365 \times 1785.77 g/mol=$ 6.51 mg have reacted with MAI. We have used 6.62 mg of spiro[TFSI]$_2$, therefore $X_2=6.51mg \times 100\%/6.62mg=98.31\%$ of the starting material have been used up in the reaction.

Experiment 3

Violet colour of the sample disappears after 0.71 ml of sodium thiosulphate solution was used for titration.

Amount of sodium thiosulphate in 250 ml of 0.01 N solution was:

m= 0.01N×124.09 g/eq×0.25l=0.310225 g

Amount of sodium thiosulphate in 0.71 ml of 0.01 N solution was:

m= 310.225mg×0.71ml/250ml=0.881 mg (0.00355 mmol)

According to the reaction equation 0.00355 mmol of spiro[TFSI]$_2$ or m=0.00355×1785.77g/mol= 6.34 mg have reacted with MAI. We have used 6.49 mg of spiro[TFSI]$_2$, therefore X_3=6.34mg×100%/6.49mg=97.68% of the starting material have been used up in the reaction.

The experiment was repeated 3 times in total and X_1= 99.52 %; X_2 = 98.31%, X_3=97.68% gives $X_{average}$= 98,49 ±0,91%, indicating good correlation between amount of spiro[TFSI]$_2$ used and iodine produced during reaction as well as proving the validity of the proposed reaction that takes place between spiro[TFSI]$_2$ and MAI.

Preparation of MAPI and CsMAFA perovskite solar cells

Conductive fluorine-doped tin oxide (FTO) coated glass substrates (Tec15, Pilkington) were used for the device fabrication. FTO substrates were cleaned with Hellmanex 2% and rinsed with water and isopropyl alcohol in an ultrasonic bath for 10 minutes each step. A compact titanium dioxide layer of about 30 nm was deposited on FTO substrates by spray pyrolysis of 4.5ml ethanol solution containing 0.3 mL titanium diisopropoxide bis(acetylacetonate) (75% in 2-propanol, Sigma-Aldrich) and 0.2mL acetylacetone (≥99%, Sigma-Aldrich) at 450°C in air. On top of this layer, approximately 300-400 nm thick mesoporous titanium dioxide was formed by spin-coating 30 nm sized TiO_2 nanoparticles (30NRT, Dyesol) diluted in ethanol (≥99.8%, Sigma-Aldrich) (1:3.5 w/w) at 4800 r.p.m. for 20 s. The formed layer was gradually heated up to 500 degrees and sintered for 1 hour in ambient atmosphere. MAPI solar cells were fabricated using two-step deposition method. Lead iodide (PbI_2) (99%, Acros) was dissolved in *N*,*N*-

dimethylformamide (99.8%, Acros) by vigorous stirring at 120° C to make 1.2 M stock solution. Lead salt was spin coated on the top of mesoporous TiO_2 layer at 6500 r.p.m. for 20 s and left for drying for 10 min at 80 degrees. The deposition of lead iodide was performed two times. On the top of lead salt, 0.05 M CH_3NH_3I in isopropanol solution was sprayed and left for 20 s before spin coating at 4000 r.p.m. for 20 s. Perovskite films were dried at 80 degrees for 15 min. CsFAMA solar cells were fabricated following the same film formation procedure described above. A hole transporting material (HTM) was deposited on the top of formed MAPI and CsFAMA perovskite films, by spin coating at 3000 rpm for 20 s. HTM was prepared by dissolving 74 mg spiro-MeOTAD in 1 ml chlorobenzene and additionally mixing it with 28.8 µL of tert-butylpyridine (96%, Sigma-Aldrich), 17.5 µL of bis(trifluoromethane)sulfonimide lithium salt (LiTFSI) (99.95% Sigma-Aldrich) (stock solution Li-TFSI 520 mg/ml in acetonitrile (99.9%, Acros)) and 29 µl of tris(2-(1H-pyrazol-1-yl)-4-tert-butylpyridine)cobalt(III) (stock solution FK-209 300 mg/ml in acetonitrile). Devices were completed by thermally evaporating 80 nm thick gold layer on the top of the HTM. For the solar cell stability experiments under different conditions, we made a batch of six devices for each composition and used two devices with the best performance for long-term experiments.

Reaction between oxidized spiro[TFSI]$_2$ and methylammonium iodide

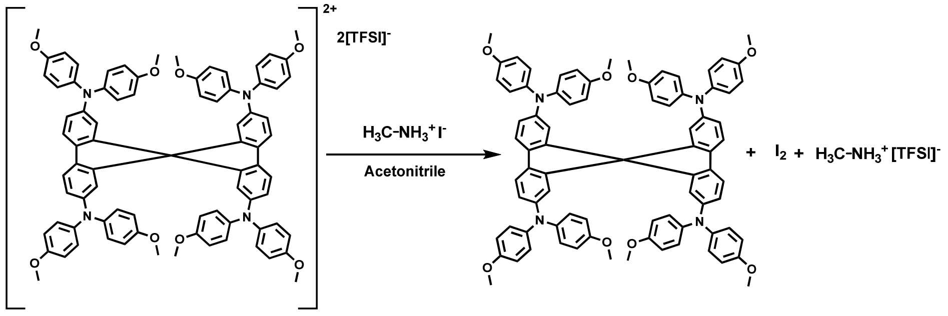

spiro[TFSI]$_2$ (0.1 g 0.056 mmol) was dissolved in acetonitrile (5 ml) then methylammonium iodide (0.09 g 0.56 mmol) was added and stirred at room temperature for 10 min . After the reaction has ended (TLC acetone: n-hexane, 2:3) precipitate was collected from solution and was purified by column chromatography (acetone: n-hexane, 1:4), yielding 0.062 g (90%) of spiro-OMeTAD.

Reaction between oxidized spiro[TFSI]$_2$ and formamidinium iodide

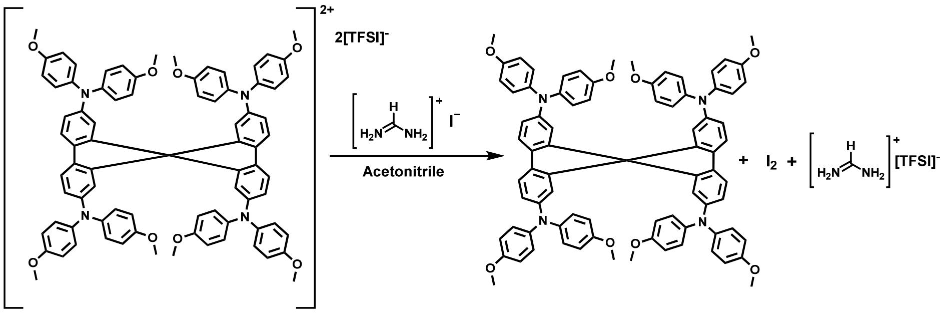

spiro[TFSI]$_2$ (0.1 g 0.056 mmol) was dissolved in acetonitrile (5 ml) then formamidinium iodide (0.05 g 0.29 mmol) was added and stirred at room temperature for 10 min . After the reaction has ended (TLC acetone: n-hexane, 2:3) precipitate was collected from solution and was purified by column chromatography (acetone: n-hexane, 1:4), yielding 0.067 g (98%) of spiro-OMeTAD.

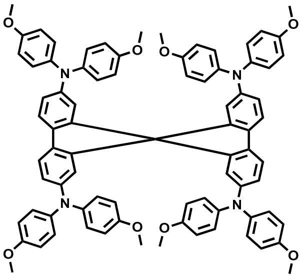

2,2',7,7'-tetrakis[*N*, *N*-di(4-methoxyphenyl)amino]-9,9'-spirobifluorene

^{1}H NMR (400 MHz, DMSO-d_6) δ 7.41 (d, *J* = 8.3 Hz, 4H), 6.81 (dd, *J* = 9.0 Hz, 32H), 6.67 (d, *J* = 8.5, 4H), 6.20 (s, 4H), 3.69 (s, 24H).

^{13}C NMR (100 MHz, DMSO-d_6) δ 155.72, 149.87, 147.63, 140.89, 134.44, 126.00, 121.35, 120.71, 116.14, 115.14, 65.45, 55.63.

MS (APCI) *m/z* [M+H]+ = 1225.

Anal. calcd for $C_{81}H_{68}N_4O_8$: C, 79.39; H, 5.59; N, 4.57; found: C, 79.21; H, 5.47; N, 4.65.

Table S1. Conductivity of the samples at room temperature

**Sample**	**Conductance (S/cm)**
CsMAFA+ spiro[TFSI]$_2$	$9{,}0\times10^{-4}$
CsMAFA+ spiro-OMeTAD	$3{,}39\times10^{-7}$
MAFA + spiro[TFSI]$_2$	$4{,}93\times10^{-4}$
MAFA + spiro-OMeTAD	$1{,}31\times10^{-7}$
MAPI + spiro[TFSI]$_2$	$3{,}67\times10^{-5}$
MAPI + spiro-OMeTAD	$3{,}27\times10^{-8}$
MAPB + spiro[TFSI]$_2$	$7{,}36\times10^{-4}$
MAPB + spiro-OMeTAD	$2{,}16\times10^{-6}$
CsFA+ spiro[TFSI]$_2$	$1{,}19\times10^{-3}$
CsFA+ spiro-OMeTAD	$2{,}58\times10^{-6}$
spiro[TFSI]$_2$	$1{,}57\times10^{-4}$
spiro-OMeTAD	$2{,}00\times10^{-8}$

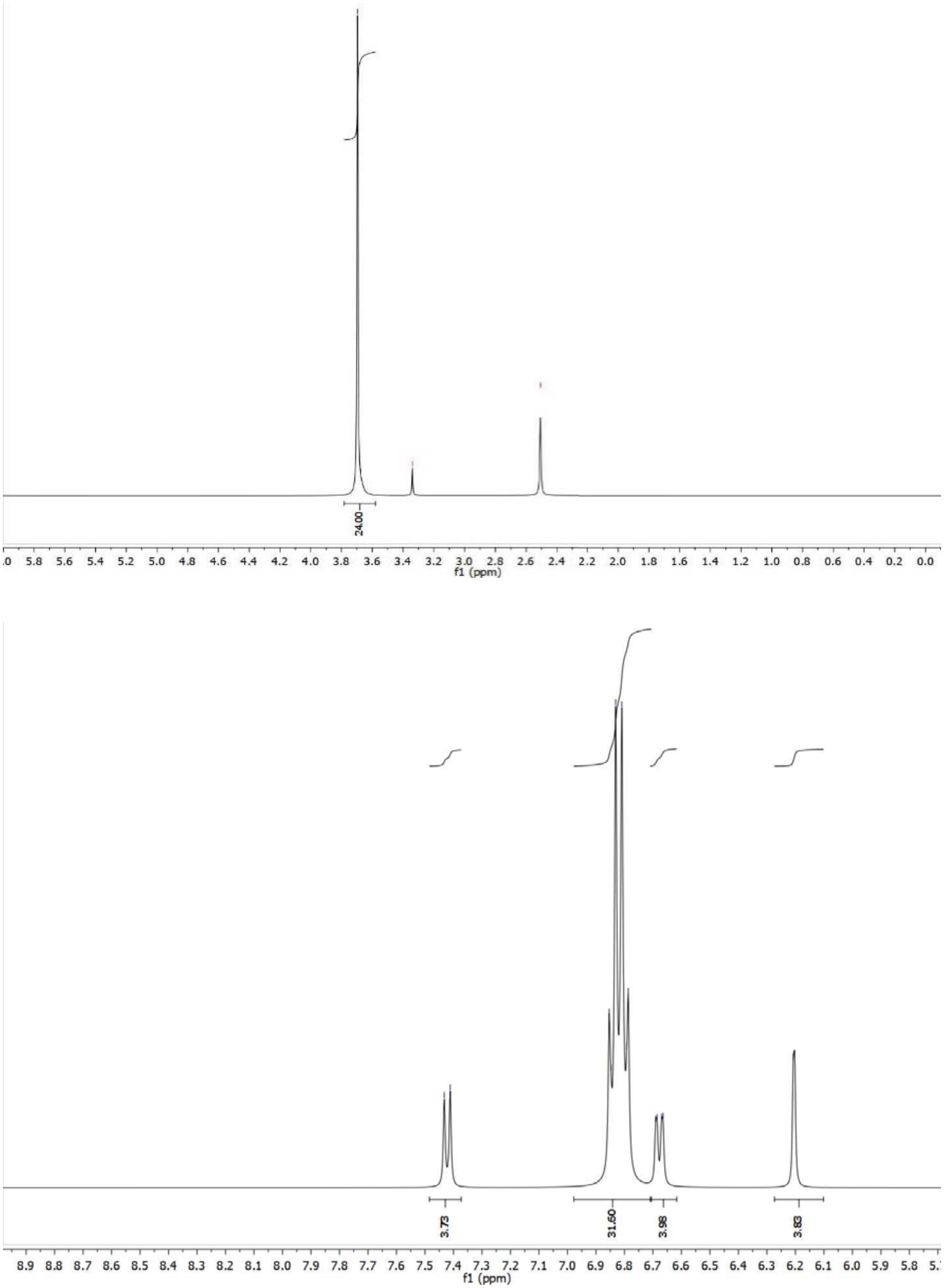

Figure S1. 1H NMR spectra of the obtained spiro-OMeTAD (400 MHz, DMSO-d_6, δ, ppm).

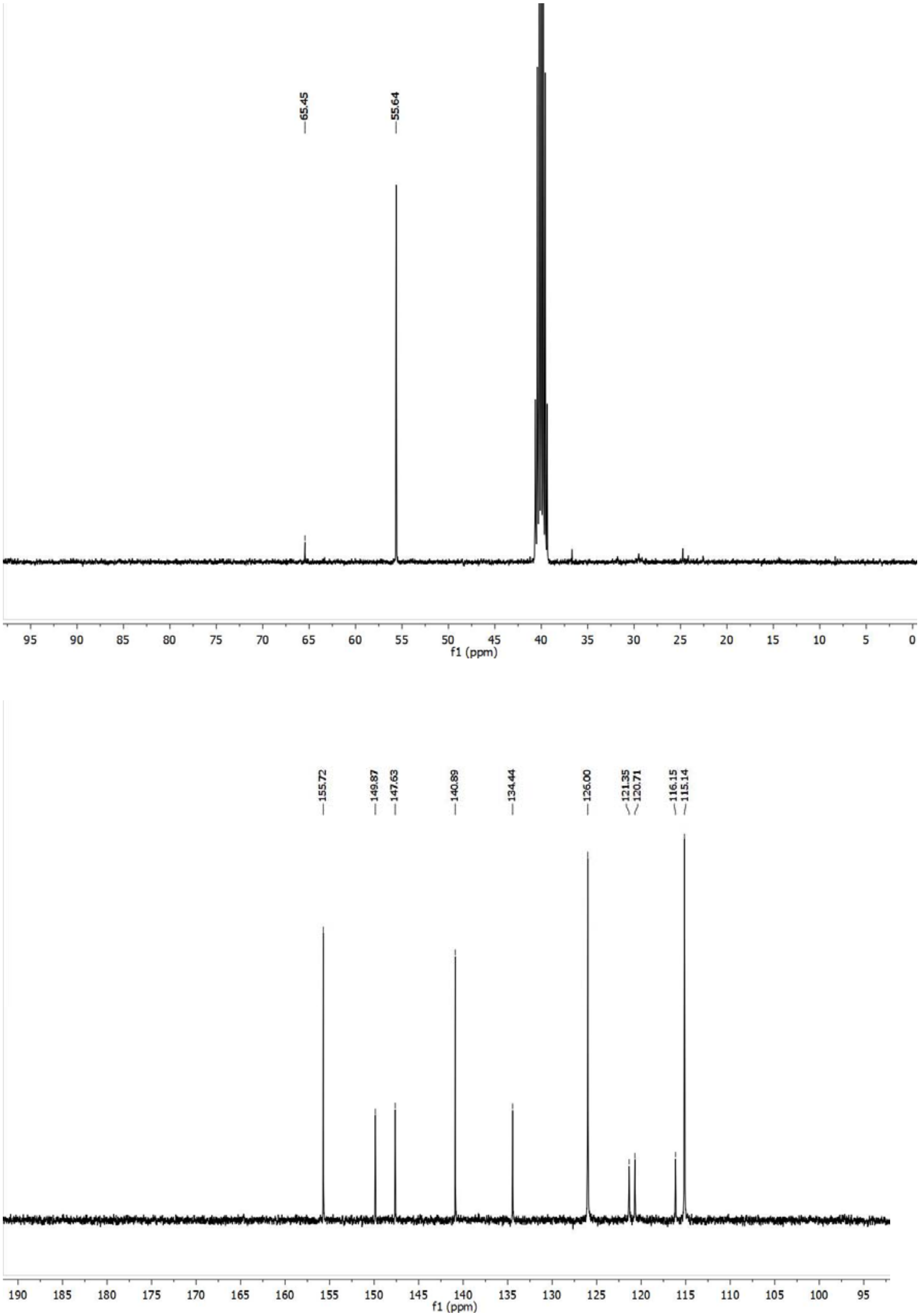

Figure S2. ^{13}C NMR spectra of the obtained spiro-OMeTAD (100 MHz, DMSO-d_6, δ, ppm).

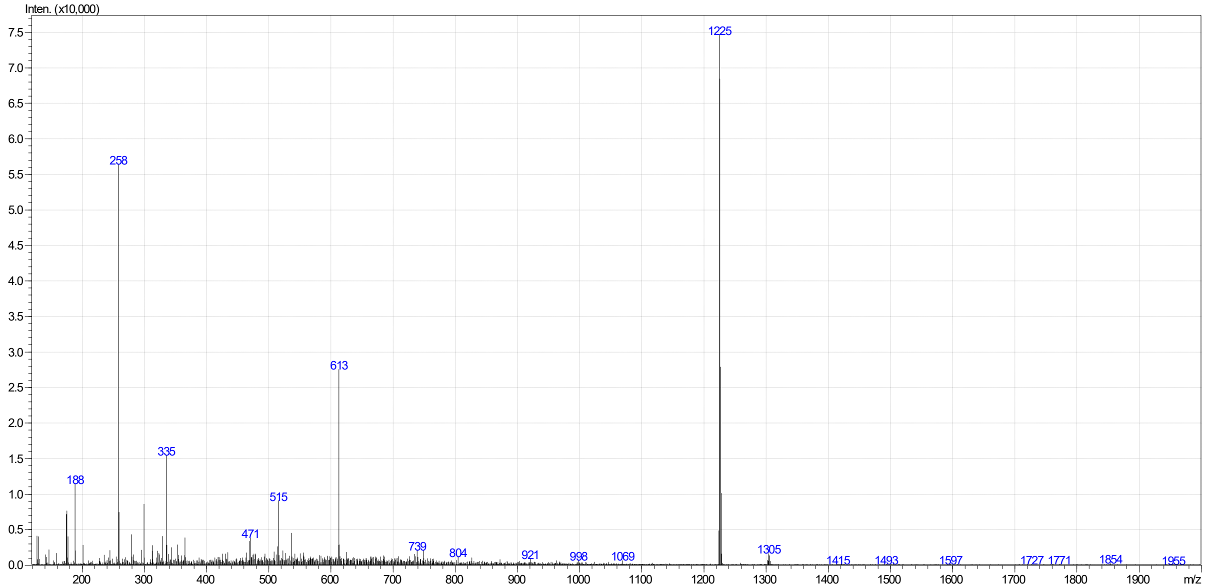

Figure S3. Mass spectra of the obtained spiro-OMeTAD after reaction (retention time 7.5 to 7.8 min).

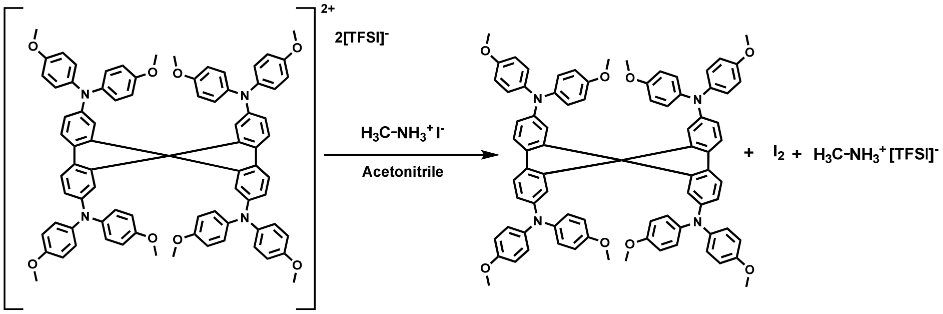

Figure S4. Proposed reaction of the oxidized spiro-OMeTAD with MAI.

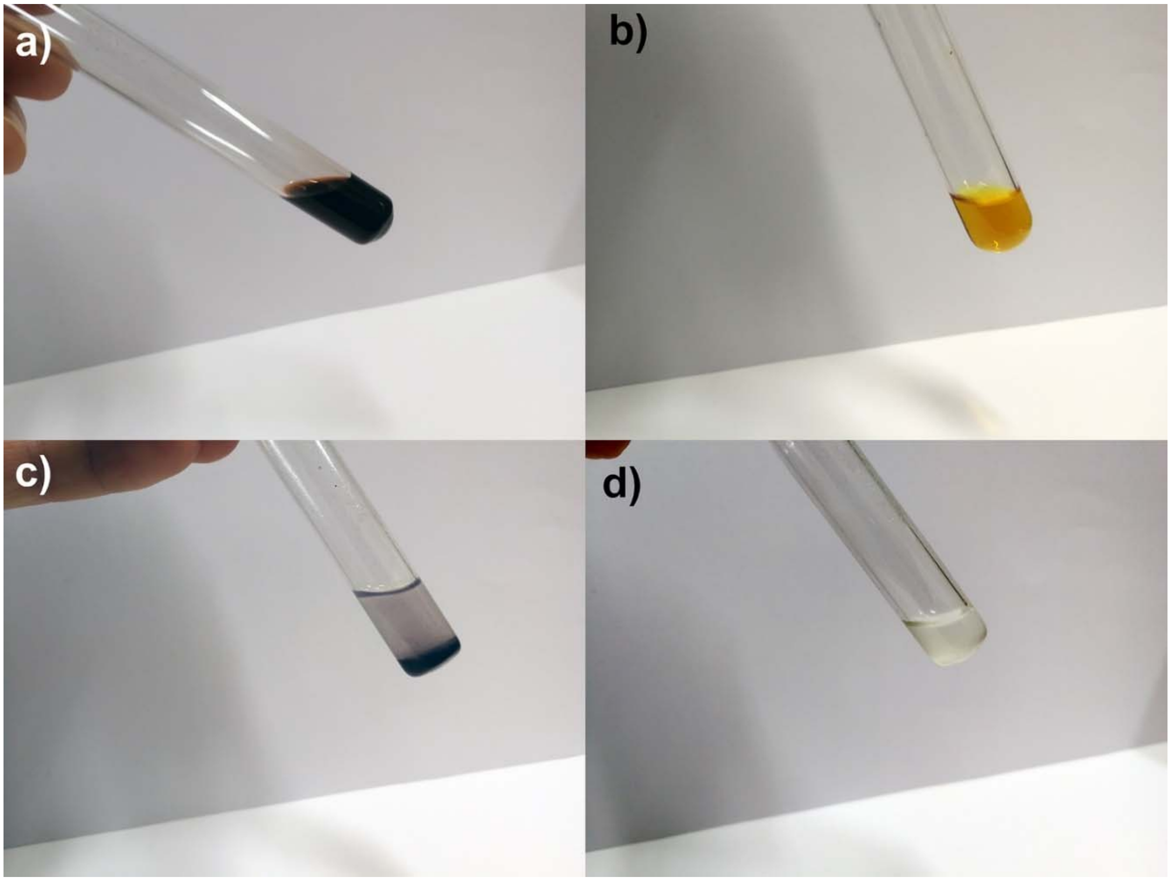

Figure S5. Detection of the iodine formed during reaction between spiro[TFSI]$_2$ and FAI, using iodine test; a) spiro[TFSI]$_2$ solution in acetonitrile; b) spiro[TFSI]$_2$ solution in acetonitrile after addition of FAI; c) spiro[TFSI]$_2$ solution in acetonitrile after addition of FAI and starch suspension in water; d) mixture of FAI and starch suspension in water.

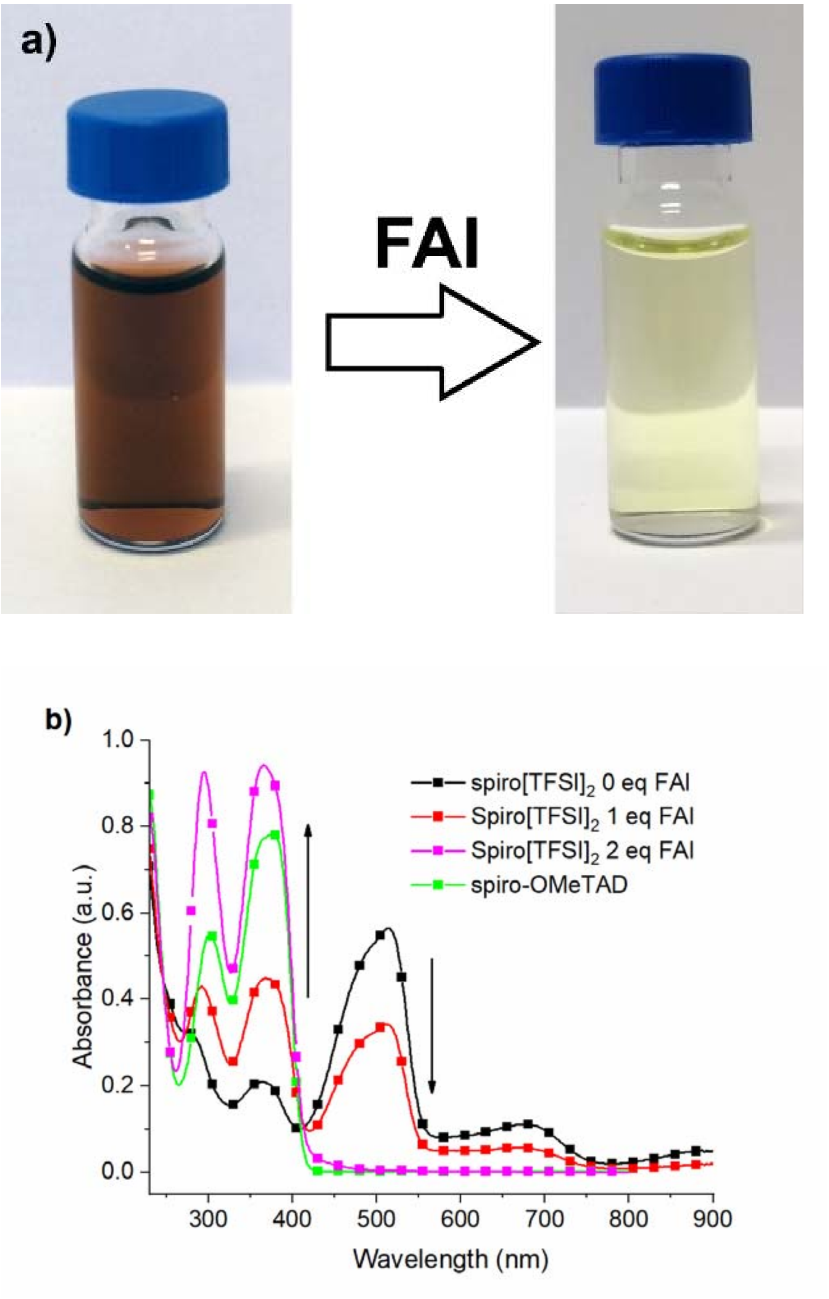

Figure S6. spiro[TFSI]$_2$ stability in acetonitrile solution (10^{-4} M) at RT after adding FAI. Photograph (a) and UV-Vis spectrum of the solution (b).

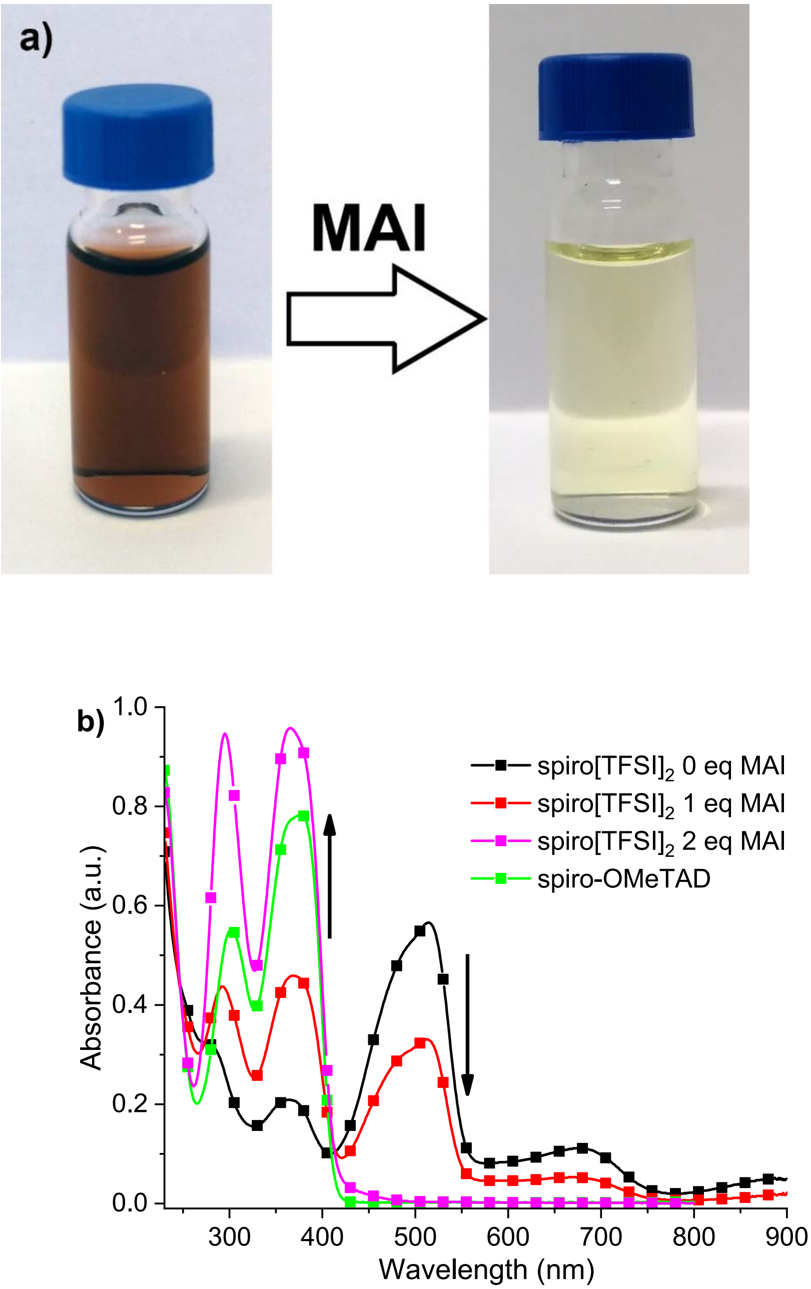

Figure S7. Stability of the diluted spiro[TFSI]$_2$ solution (10^{-4} M) in acetonitrile at RT after adding MAI. Photograph (a) and UV-Vis spectrum of the solution (b).

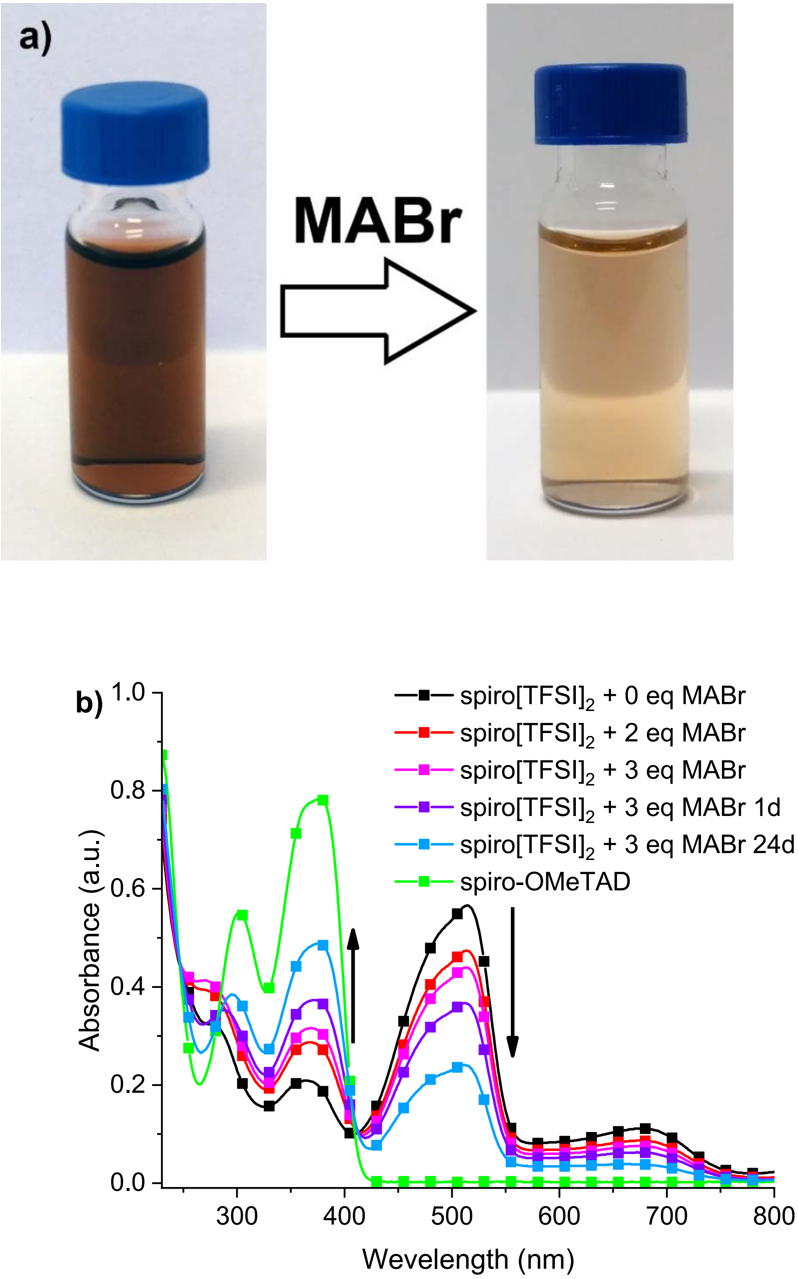

Figure S8. Stability of the diluted spiro[TFSI]$_2$ solution (10^{-4} M) in acetonitrile at RT after adding MABr. Photograph (a) and UV-Vis spectrum of the solution (b).

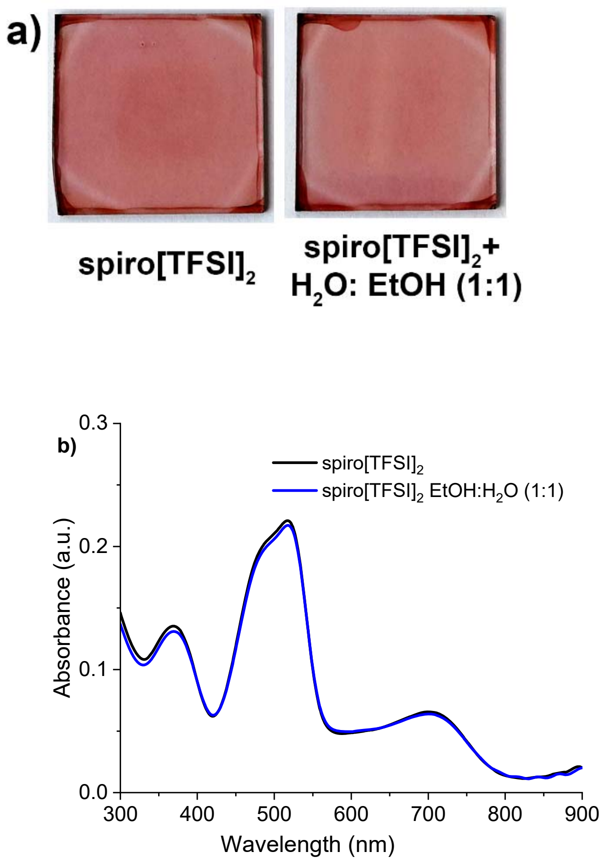

Figure S9. Photograph (a) and UV-Vis spectrum (b) of the spiro[TFSI]$_2$ film before and after interaction with solvent used in the experiment.

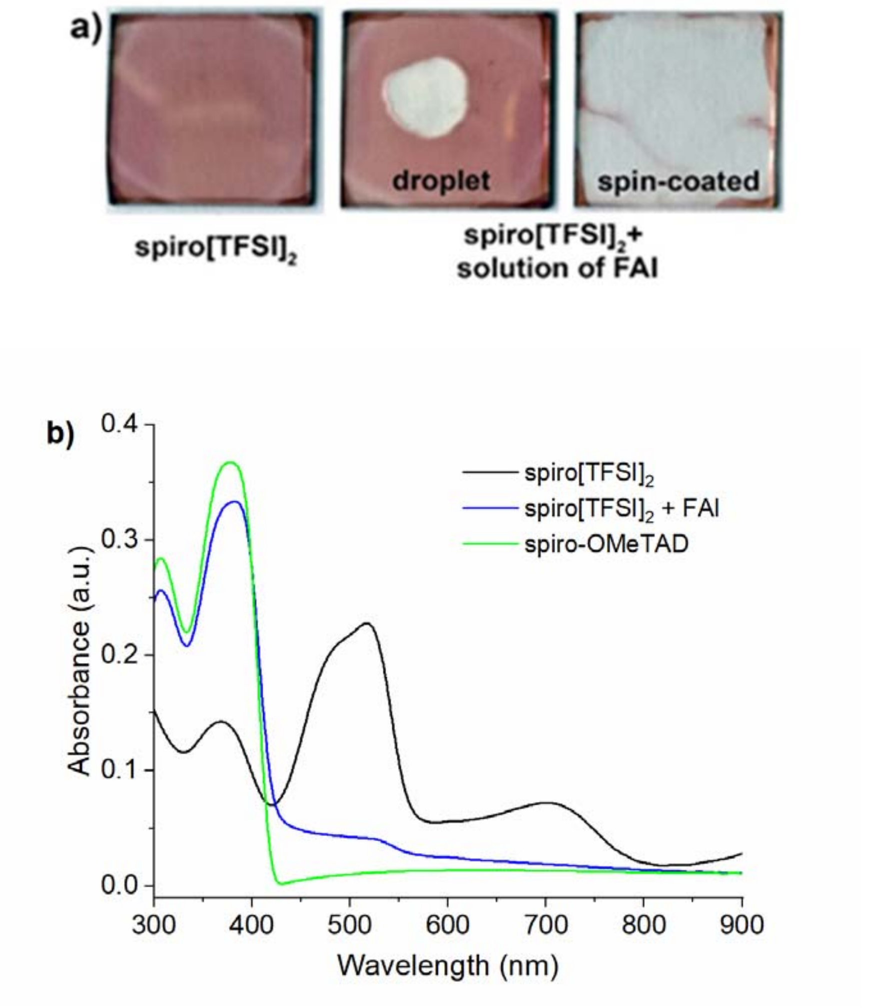

Figure S10**.** Photographs (a) and UV-Vis spectrum (b) of the spiro[TFSI]$_2$ film before and after interaction with FAI solution.

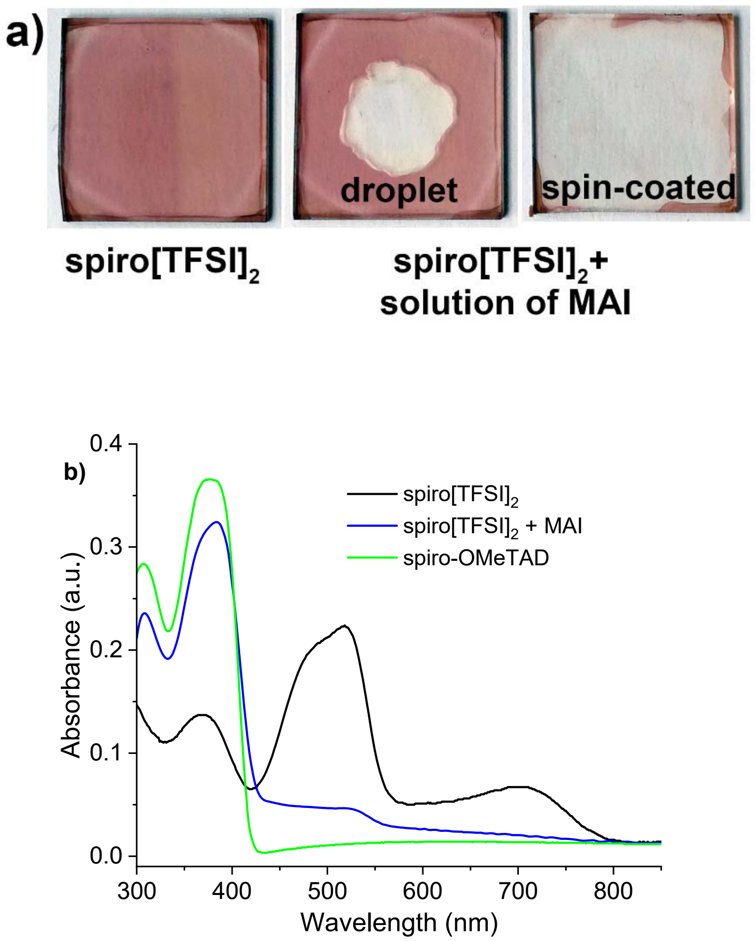

Figure S11. Photograph (a) and UV-Vis spectrum (b) of the spiro$[TFSI]_2$ film before and after interaction with MAI solution.

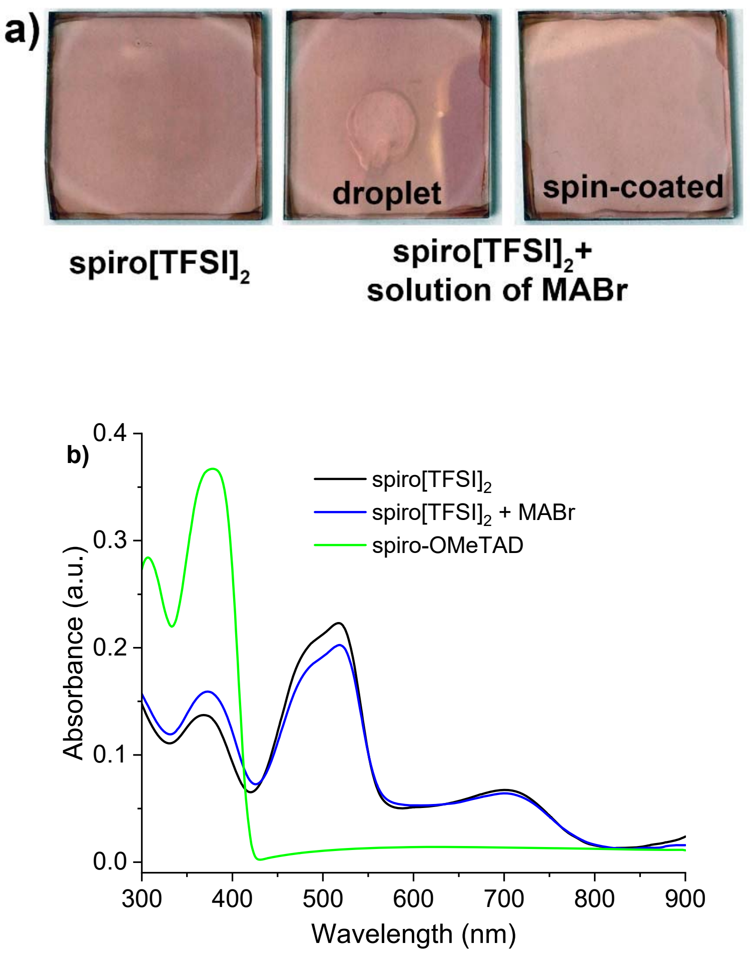

Figure S12. Photograph (a) and UV-Vis spectrum (b) of the spiro[TFSI]$_2$ film before and after interaction with MABr solution.

Experiments with MAPI perovskite

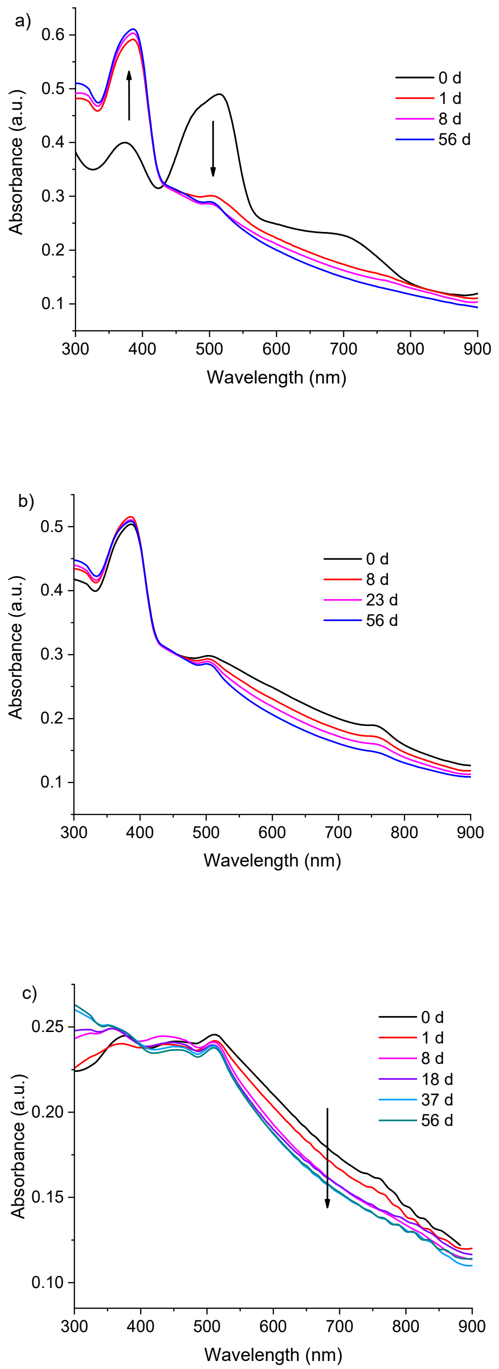

Figure S13. Stability of the encapsulated spiro[TFSI]$_2$ (a), spiro-OMeTAD (b) films on MAPI perovskite as well as pristine perovskite (c) at 100 ºC in the dark.

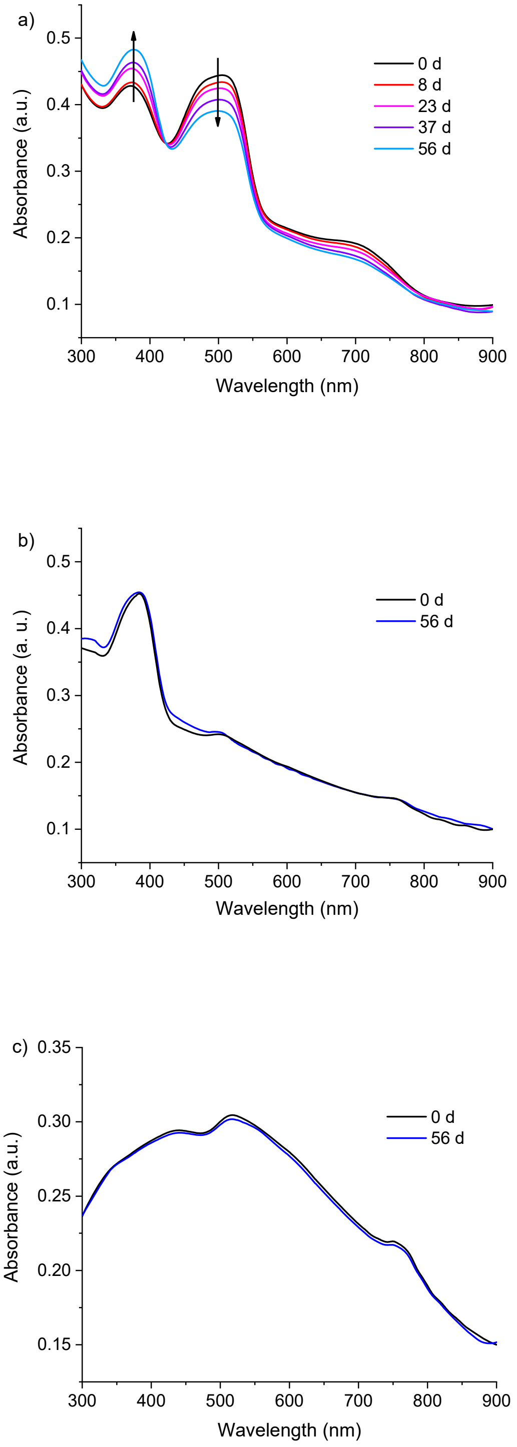

Figure S14. Stability of the encapsulated spiro[TFSI]$_2$ (a), spiro-OMeTAD (b) films on MAPI perovskite as well as pristine perovskite (c) at RT in the dark.

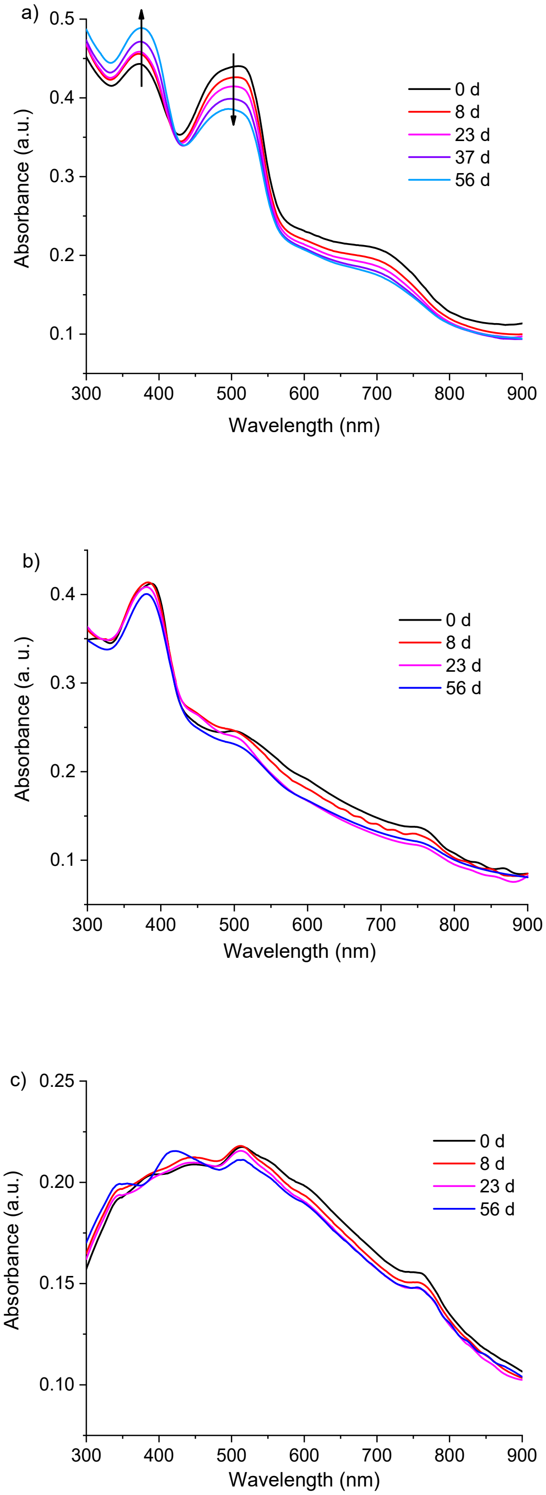

Figure S15. Stability of the encapsulated spiro[TFSI]$_2$ (a), spiro-OMeTAD (b) films on MAPI perovskite as well as pristine perovskite (c) at RT under ambient light.

Experiments with MAFA perovskite

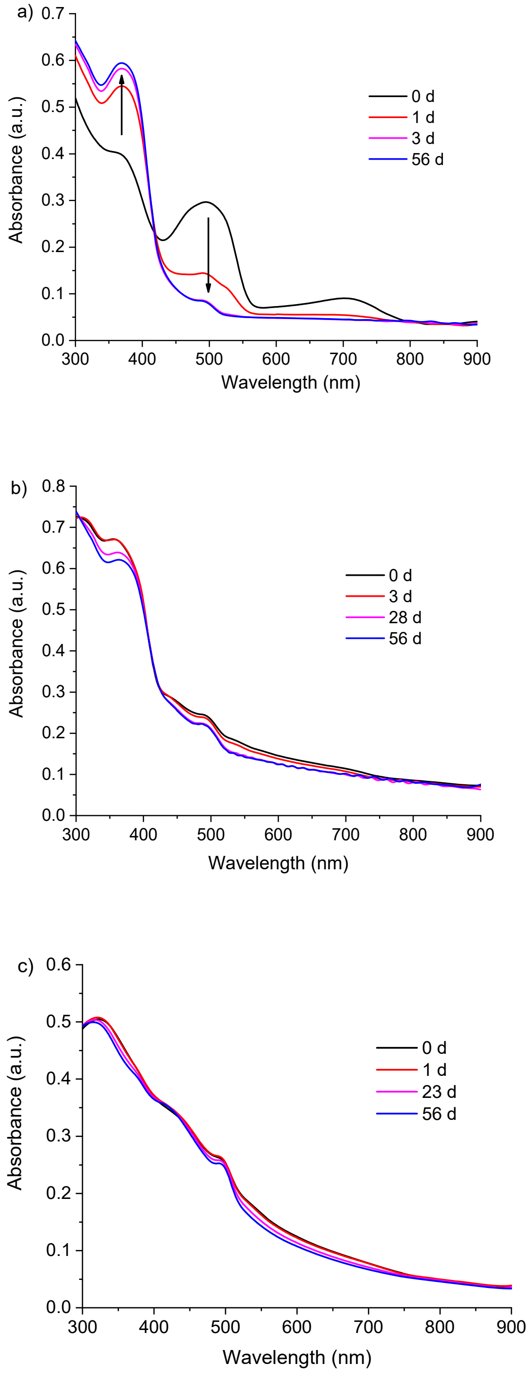

Figure S16. Stability of the encapsulated spiro[TFSI]$_2$ (a), spiro-OMeTAD (b) films on MAFA perovskite as well as pristine perovskite (c) at 100 ºC in the dark.

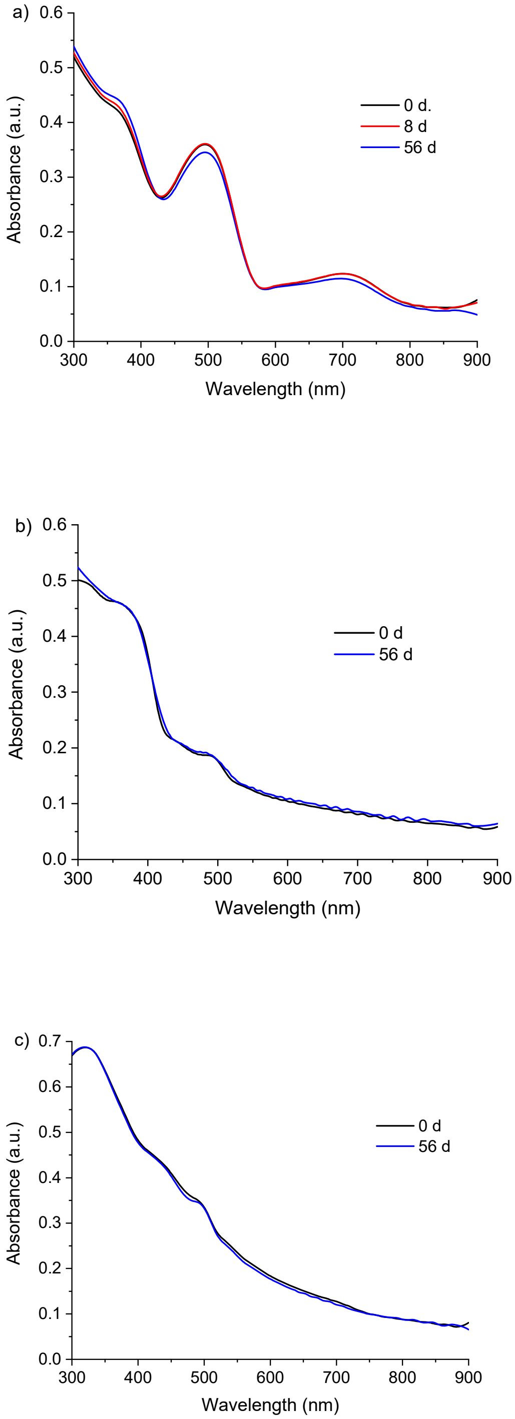

Figure S17. Stability of the encapsulated spiro[TFSI]$_2$ (a), spiro-OMeTAD (b) films on MAFA perovskite as well as pristine perovskite (c) at RT in the dark.

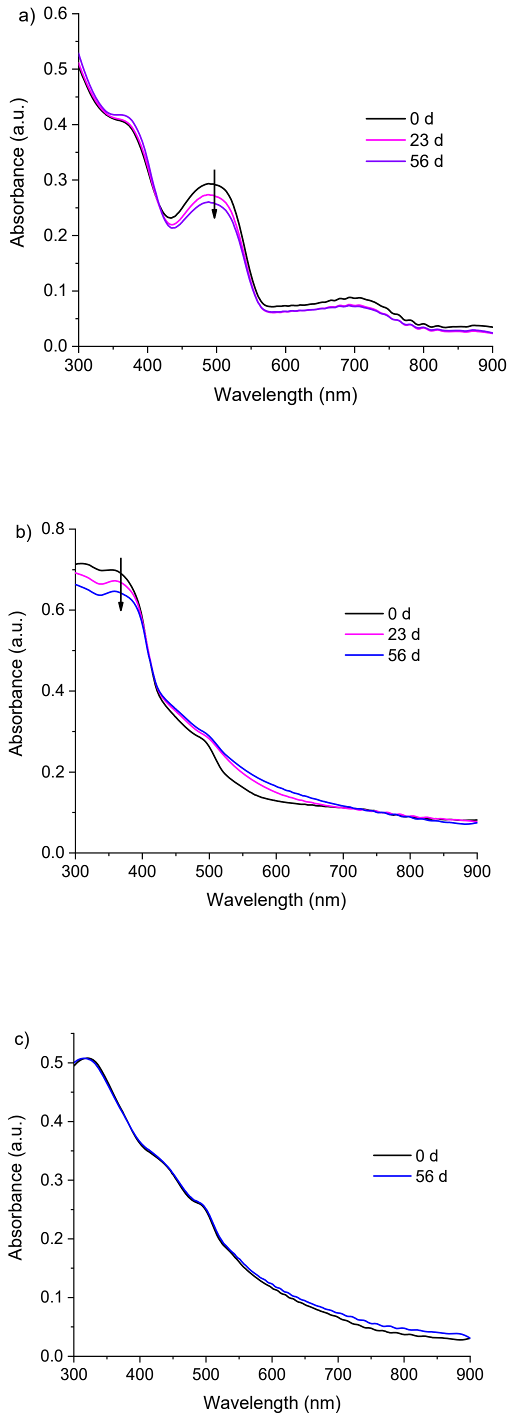

Figure S18. Stability of the encapsulated spiro[TFSI]$_2$ (a), spiro-OMeTAD (b) films on MAFA perovskite as well as pristine perovskite (c) at RT under ambient light.

Experiments with CsMAFA perovskite

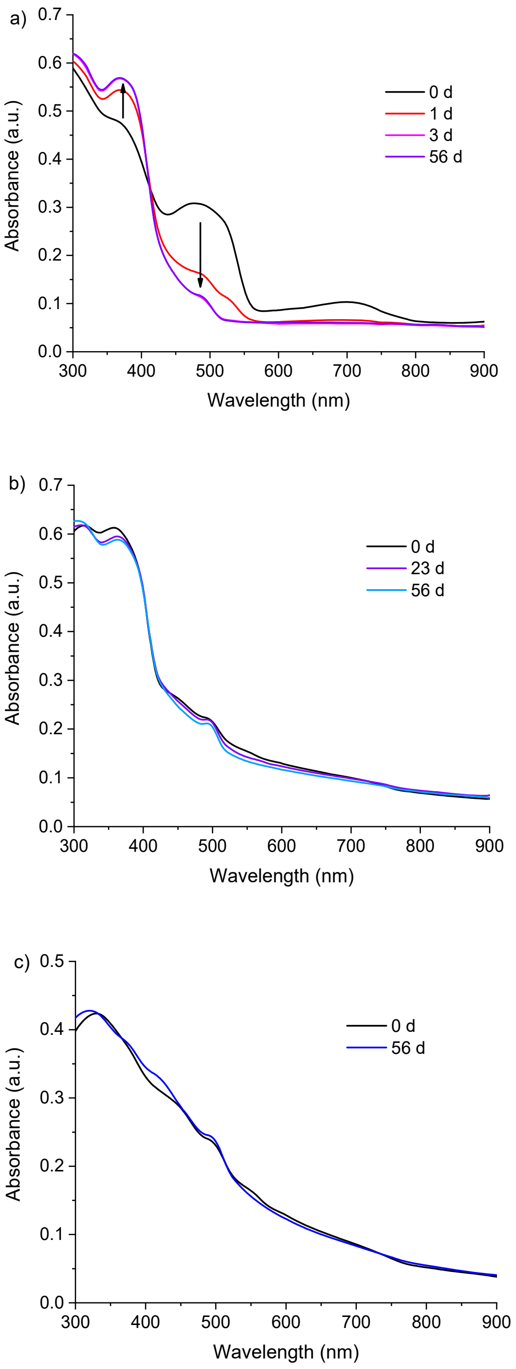

Figure S19. Stability of the encapsulated spiro[TFSI]$_2$ (a), spiro-OMeTAD (b) films on CsMAFA perovskite as well as pristine perovskite (c) at 100 ºC in the dark.

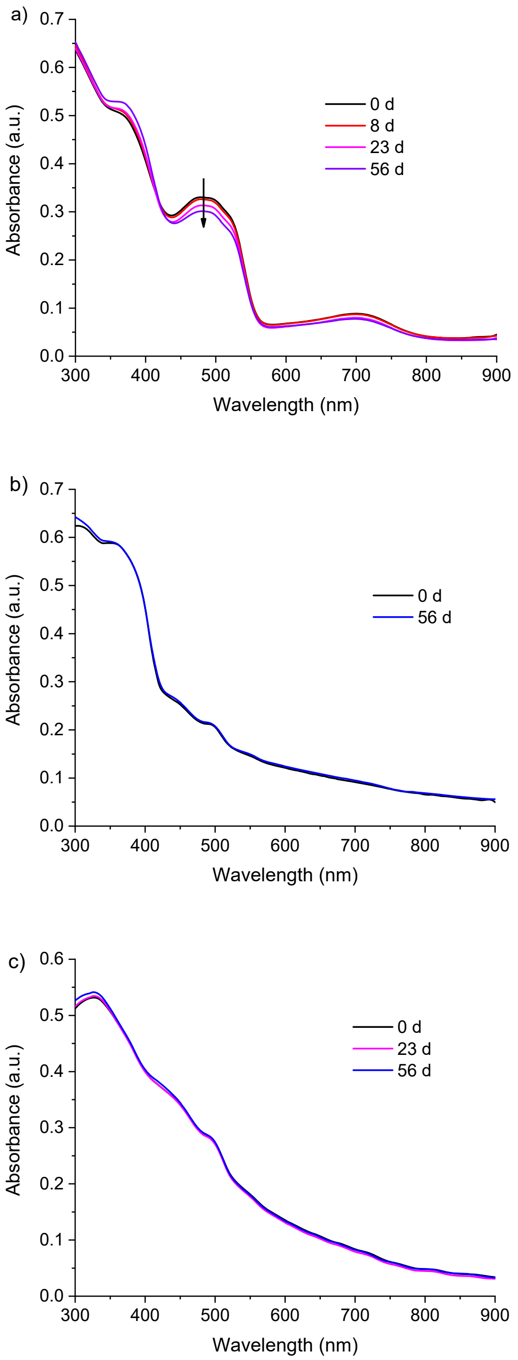

Figure S20. Stability of the encapsulated spiro[TFSI]$_2$ (a), spiro-OMeTAD (b) films on CsMAFA perovskite as well as pristine perovskite (c) at RT in the dark.

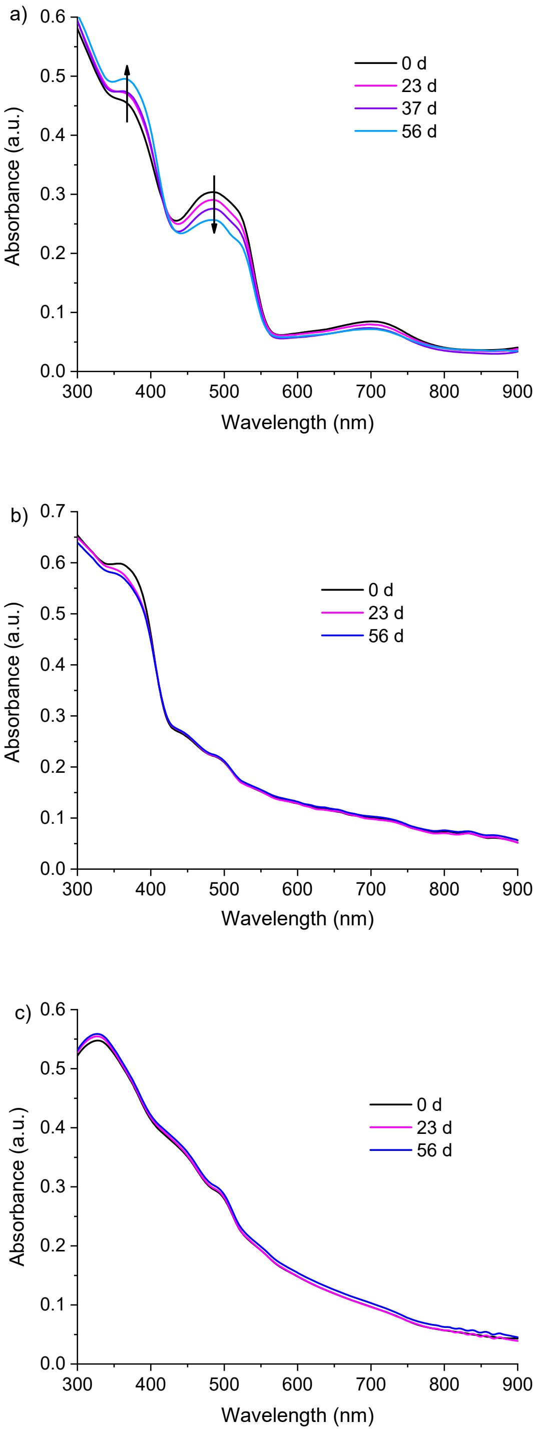

Figure S21. Stability of the encapsulated spiro[TFSI]$_2$ (a), spiro-OMeTAD (b) films on CsMAFA perovskite as well as pristine perovskite (c) at RT under ambient light.

Experiments with CsFA perovskite

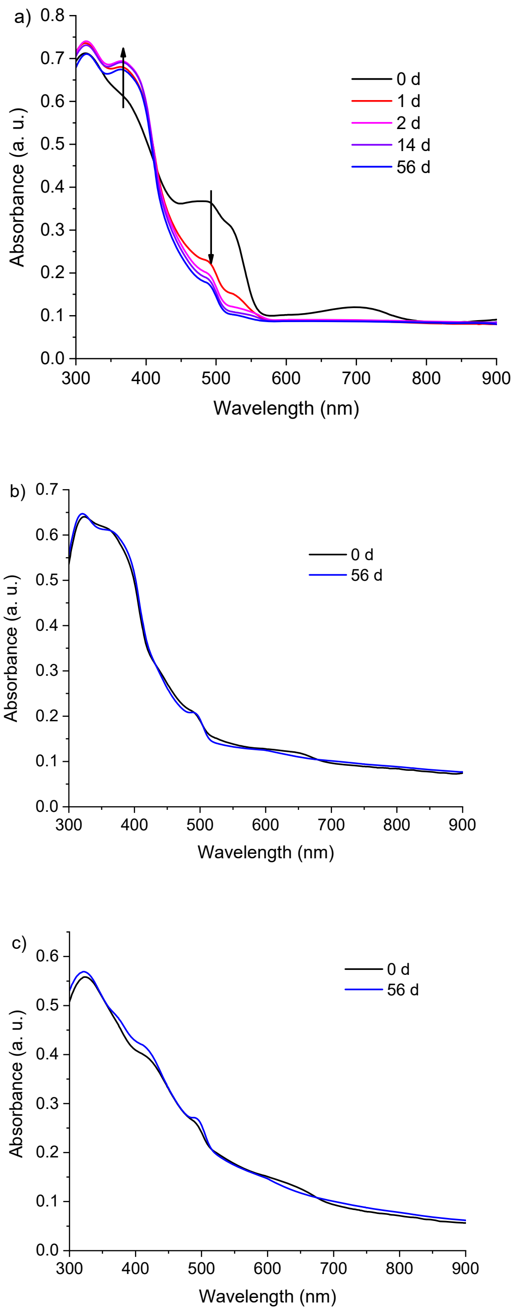

Figure S22. Stability of the encapsulated spiro[TFSI]$_2$ (a), spiro-OMeTAD (b) films on CsFA perovskite as well as pristine perovskite (c) at 100 ºC in the dark.

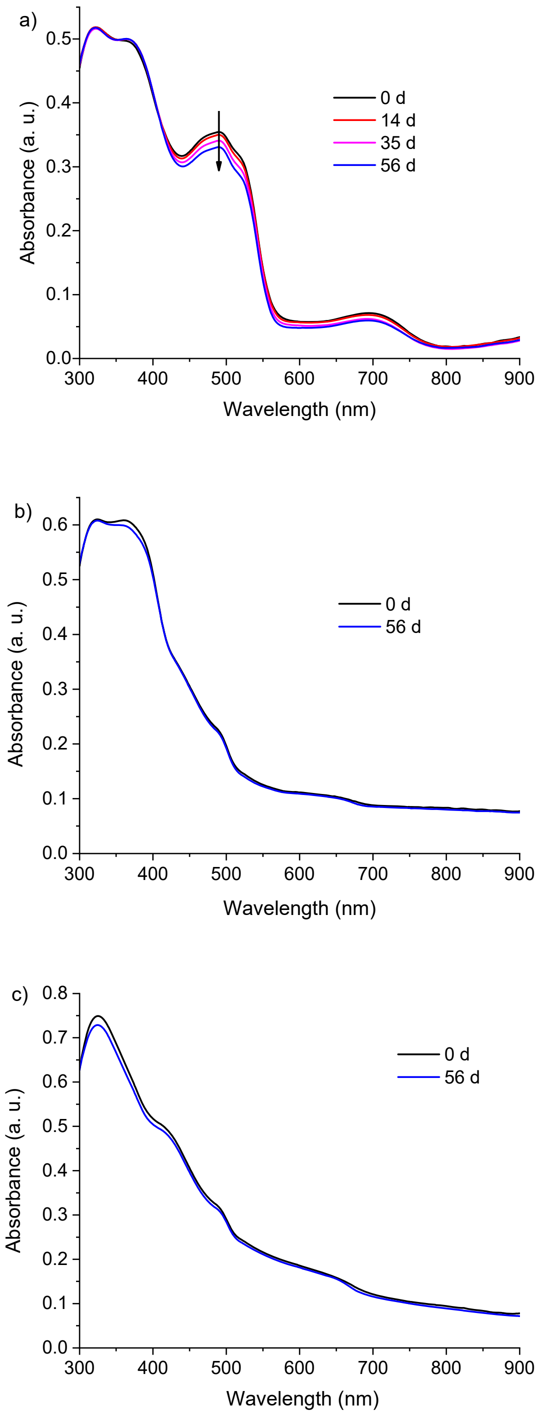

Figure S23. Stability of the encapsulated spiro[TFSI]$_2$ (a), spiro-OMeTAD (b) films on CsFA perovskite as well as pristine perovskite (c) at RT in the dark.

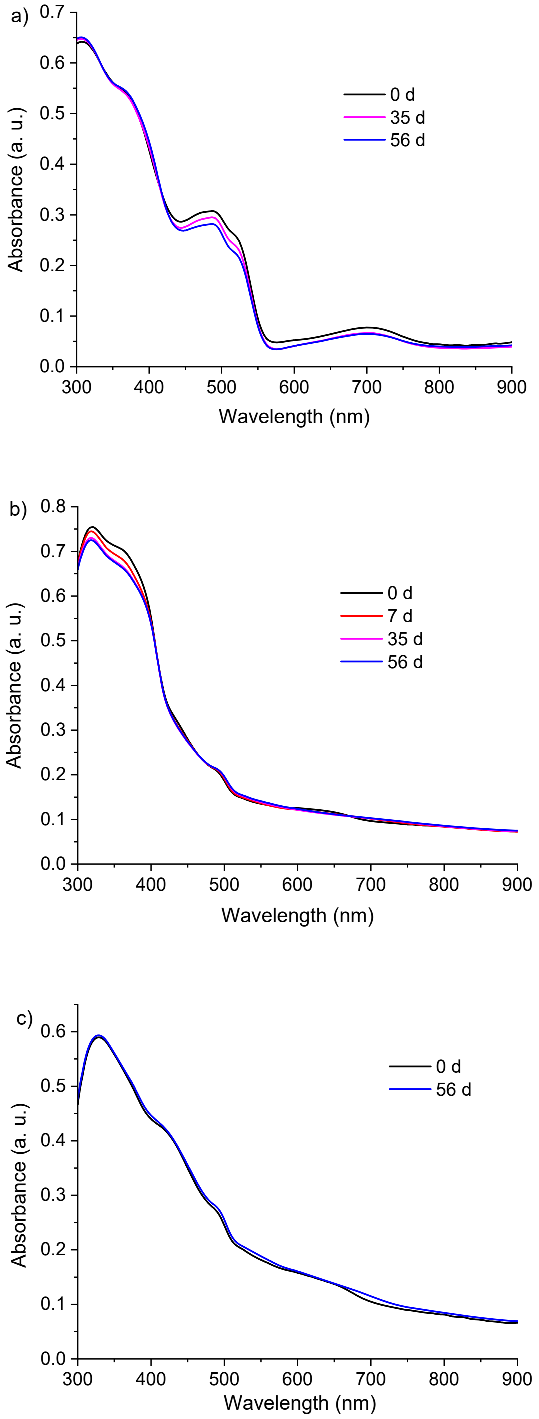

Figure S24. Stability of the encapsulated spiro[TFSI]$_2$ (a), spiro-OMeTAD (b) films on CsFA perovskite as well as pristine perovskite (c) at RT under ambient light.

Experiments with MAPB perovskite

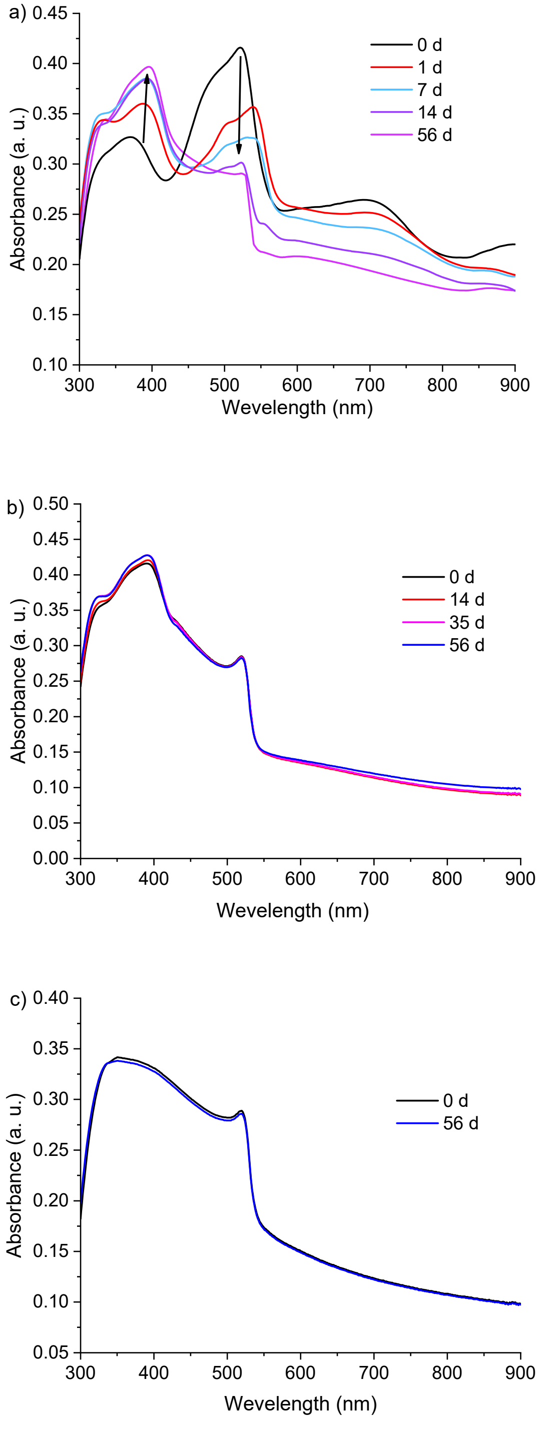

Figure S25. Stability of the encapsulated spiro[TFSI]$_2$ (a), spiro-OMeTAD (b) films on MAPB perovskite as well as pristine perovskite (c) at 100 ºC in the dark.

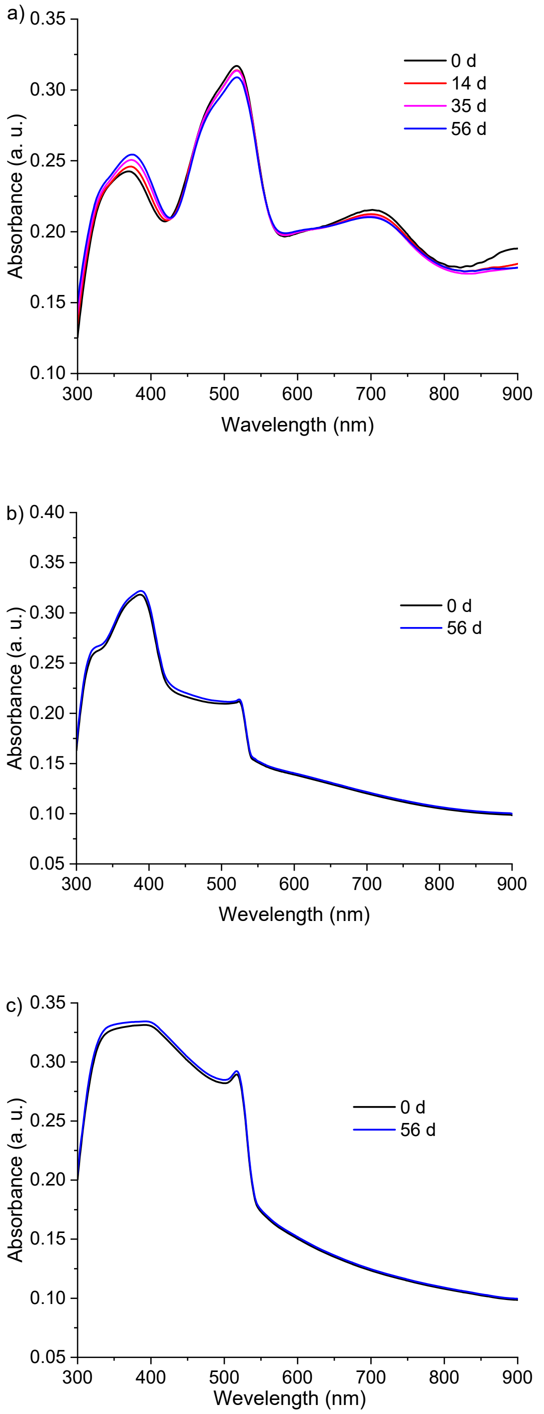

Figure S26. Stability of the encapsulated spiro[TFSI]$_2$ (a), spiro-OMeTAD (b) films on MAPB perovskite as well as pristine perovskite (c) at RT in the dark.

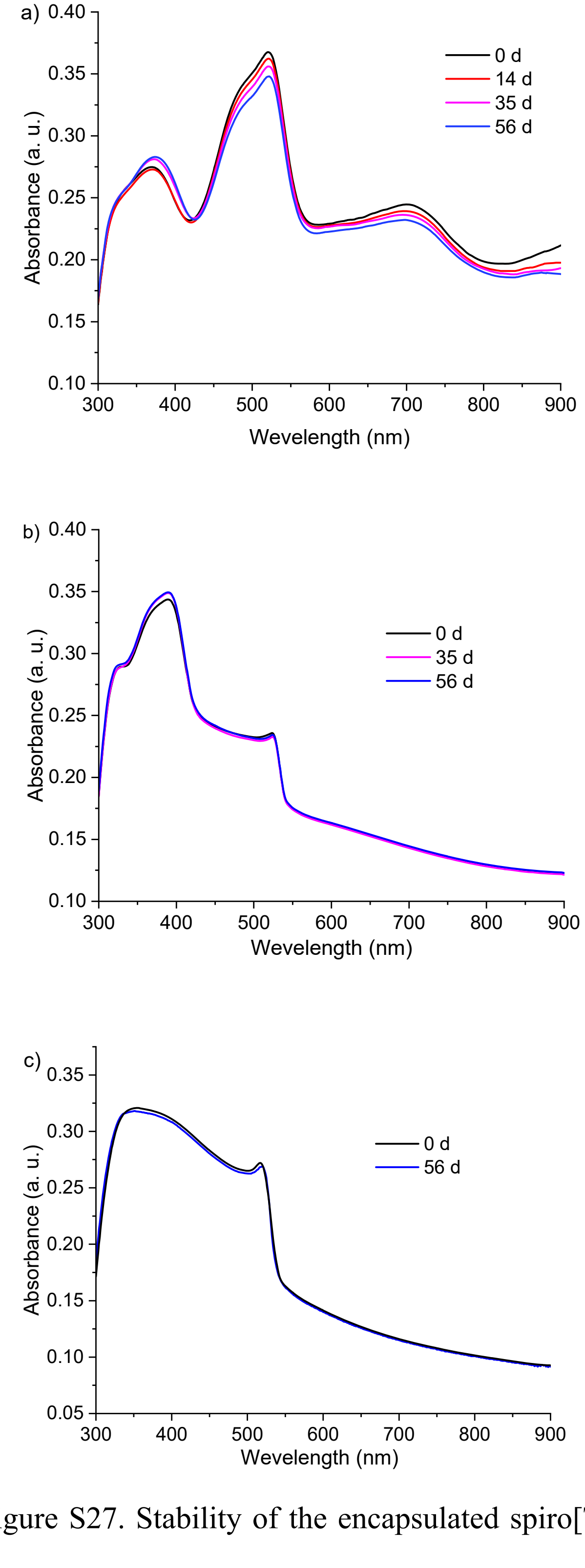

Figure S27. Stability of the encapsulated spiro[TFSI]$_2$ (a), spiro-OMeTAD (b) films on MAPB perovskite as well as pristine perovskite (c) at RT under ambient light.

MAPI	MAFA
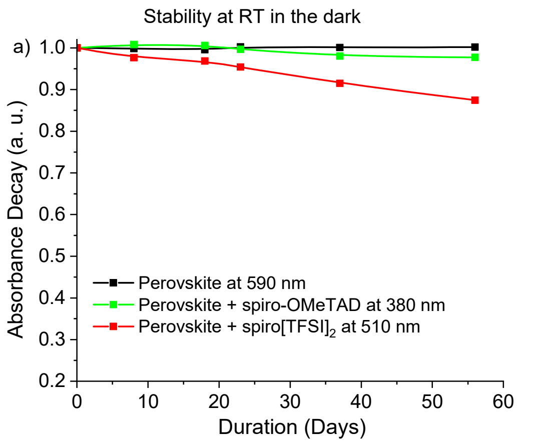	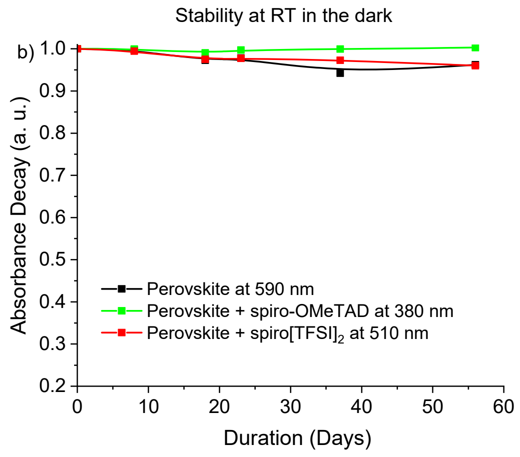

Figure S28. Light absorption intensity dynamics of the spiro[TFSI]2 or spiro-OMeTAD films on $MAPbI_3$ and $MA_{0.17}FA_{0.83}Pb(I_{0.83}Br_{0.17})_3$ perovskites.

CsMAFA	CsFA
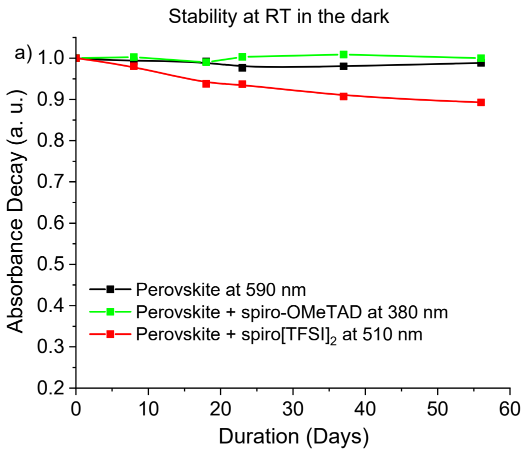	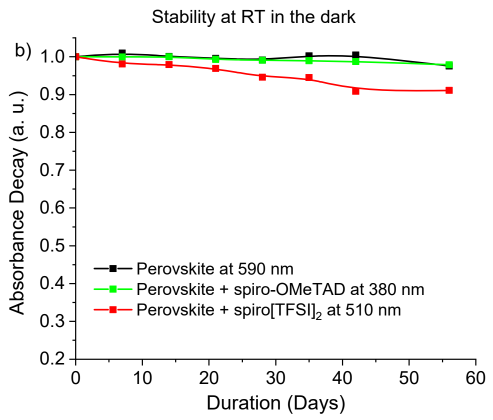

Figure S29. Light absorption intensity dynamics of the spiro[TFSI]2 or spiro-OMeTAD films on CsMAFA and CsFA perovskites.

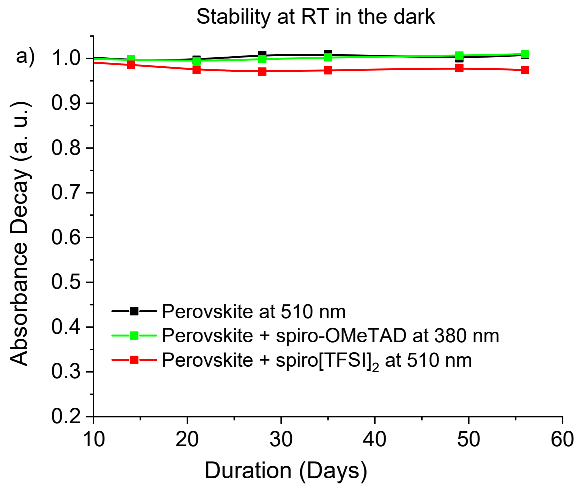

Figure S30. Light absorption intensity dynamics of the spiro[TFSI]$_2$ or spiro-OMeTAD films on MAPB perovskite.

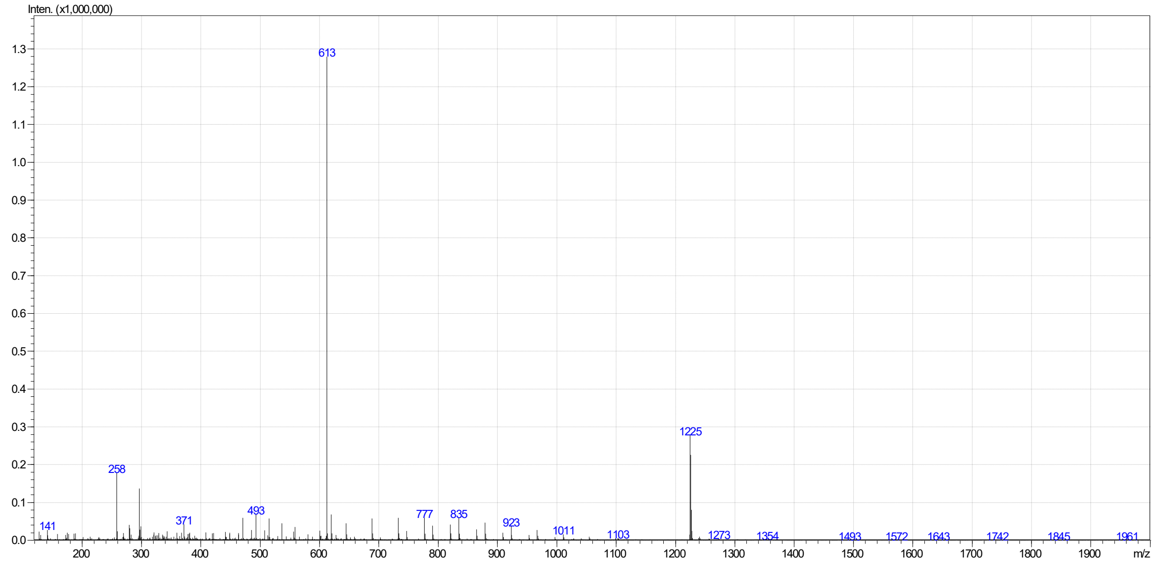

Figure S31. Mass spectra of the first fraction (retention time 6.6 to 6.95 min) detected in the sample prepared from mixture of MABr and spiro[TFSI]$_2$ in acetonitrile, kept at RT for 24 days in the dark.

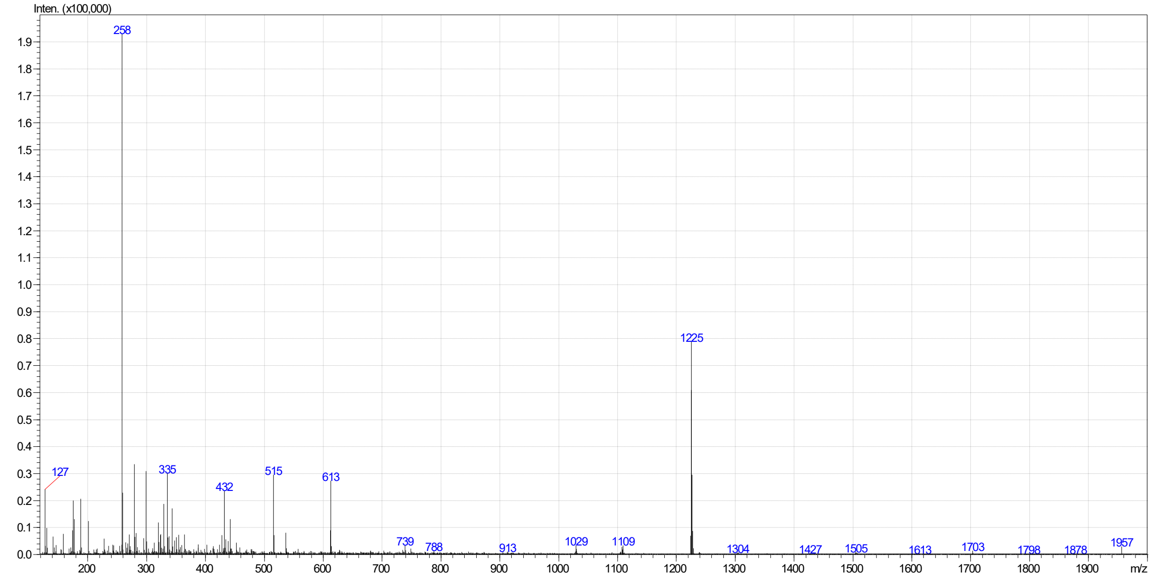

Figure S32. Mass spectra of the second fraction (retention time 7.5 to 7.8 min) detected in the sample prepared from mixture of MABr and spiro[TFSI]$_2$ in acetonitrile, kept at RT for 24 days in the dark.

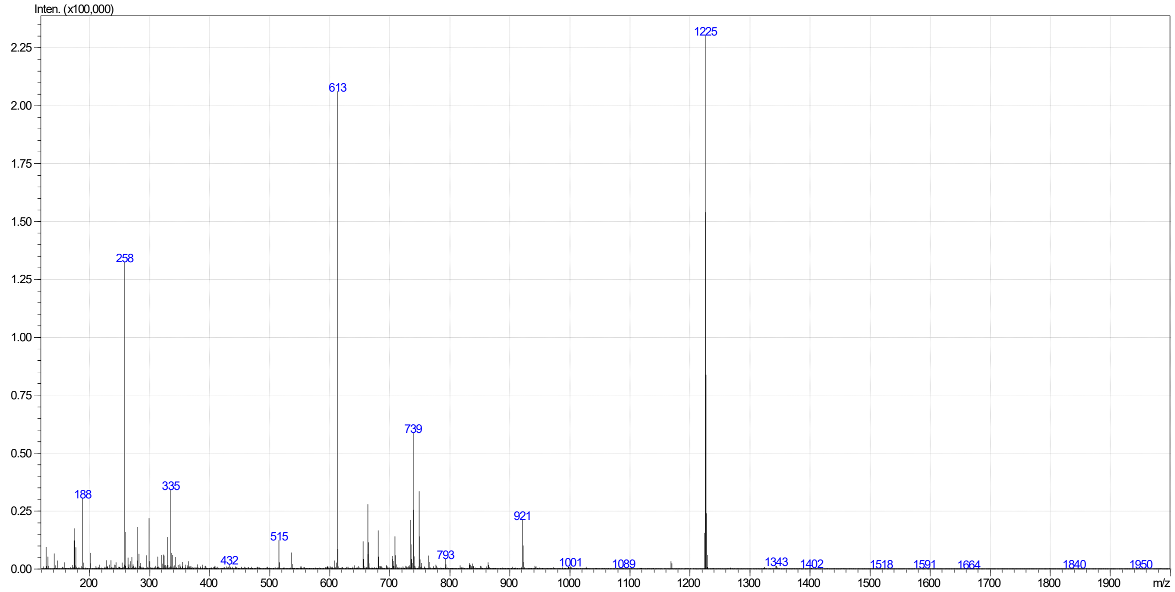

Figure S33. Mass spectra of the first fraction (retention time 7.5 to 7.8 min) detected in the sample prepared from mixture of FAI and spiro[TFSI]$_2$ in acetonitrile, kept at RT for 6 hours in the dark.

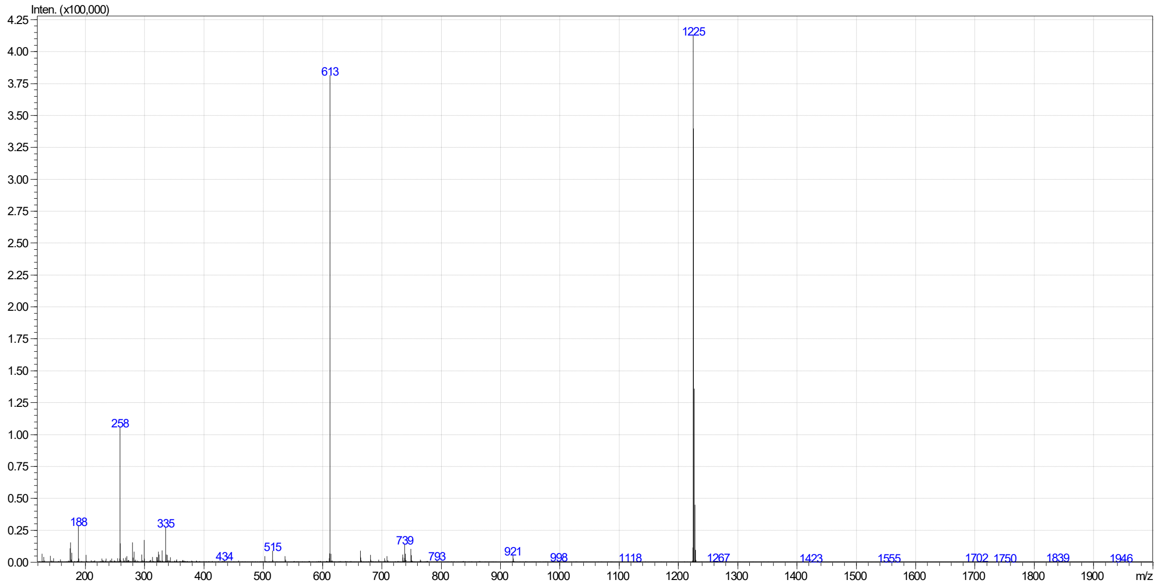

Figure S34. Mass spectra of the first fraction (retention time 7.5 to 7.8 min) detected in the sample prepared from mixture of MAI and spiro[TFSI]$_2$ in acetonitrile, kept at RT for 6 hours in the dark.

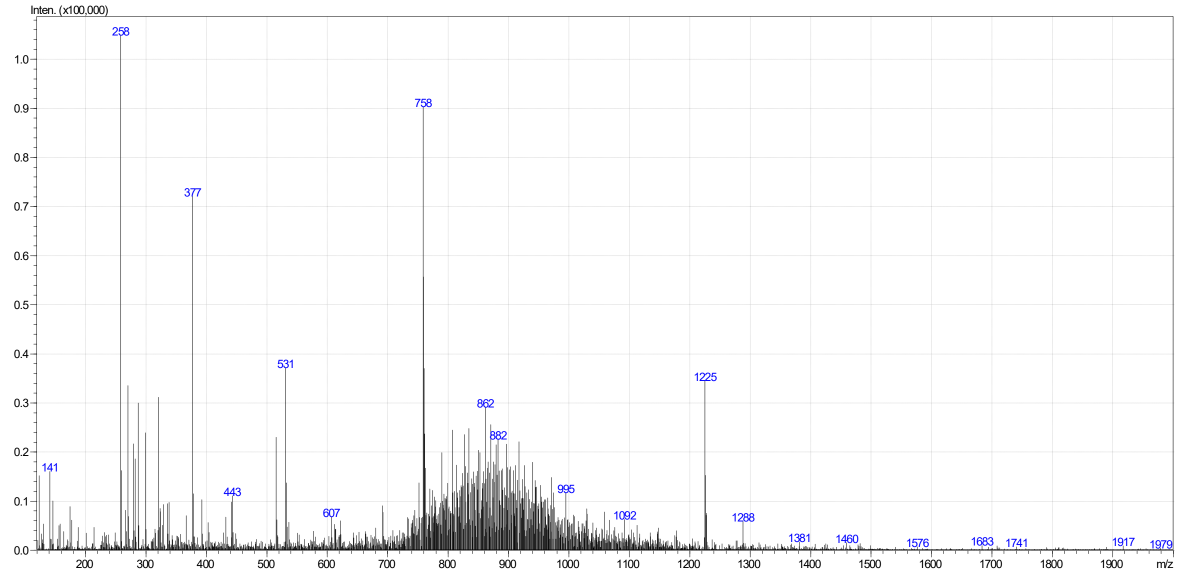

Figure S35. Mass spectra of the fraction (retention time 7.5 to 7.8 min) detected in the sample prepared from encapsulated film containing $Cs_5(MA_{0.17}FA_{0.83})_{95}Pb(I_{0.83}Br_{0.17})_3$ and spiro[TFSI]$_2$ kept at 100 °C for 56 days.

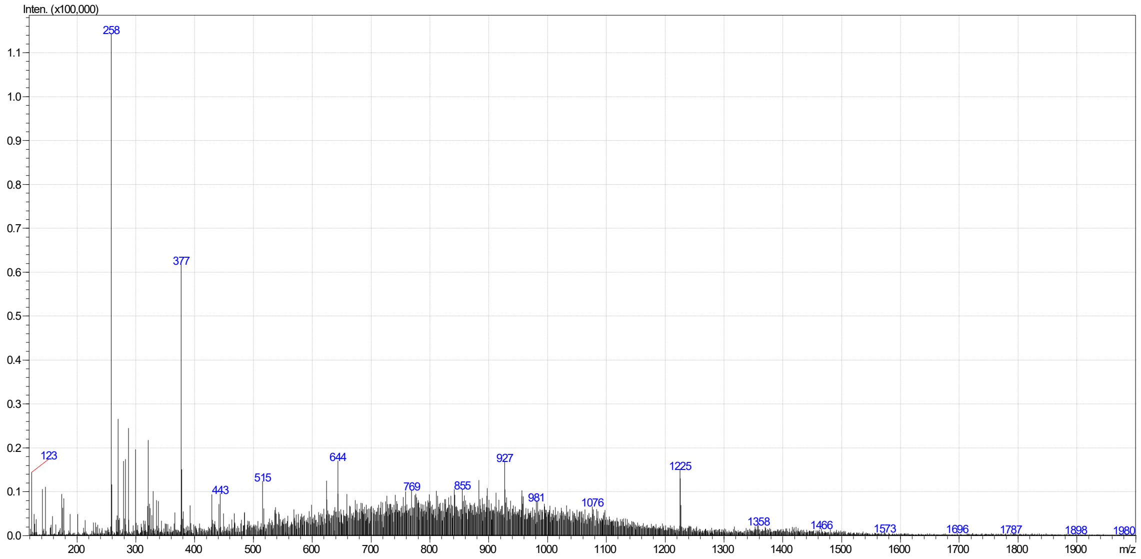

Figure S36. Mass spectra of the fraction (retention time 7.5 to 7.8 min) detected in the sample prepared from encapsulated film containing $MA_{0.17}FA_{0.83}Pb(I_{0.83}Br_{0.17})_3$ and spiro[TFSI]$_2$ kept at 100 °C for 56 days.

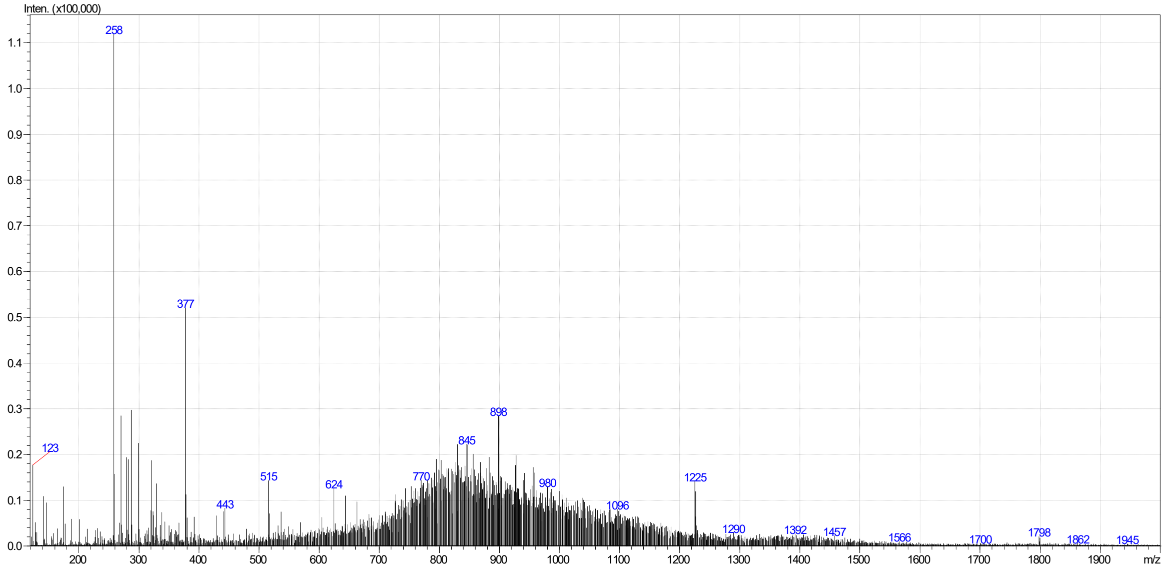

Figure S37. Mass spectra of the fraction (retention time 7.5 to 7.8 min) detected in the sample prepared from encapsulated film containing MAPbI3 and spiro[TFSI]$_2$ kept at 100 °C for 56 days.

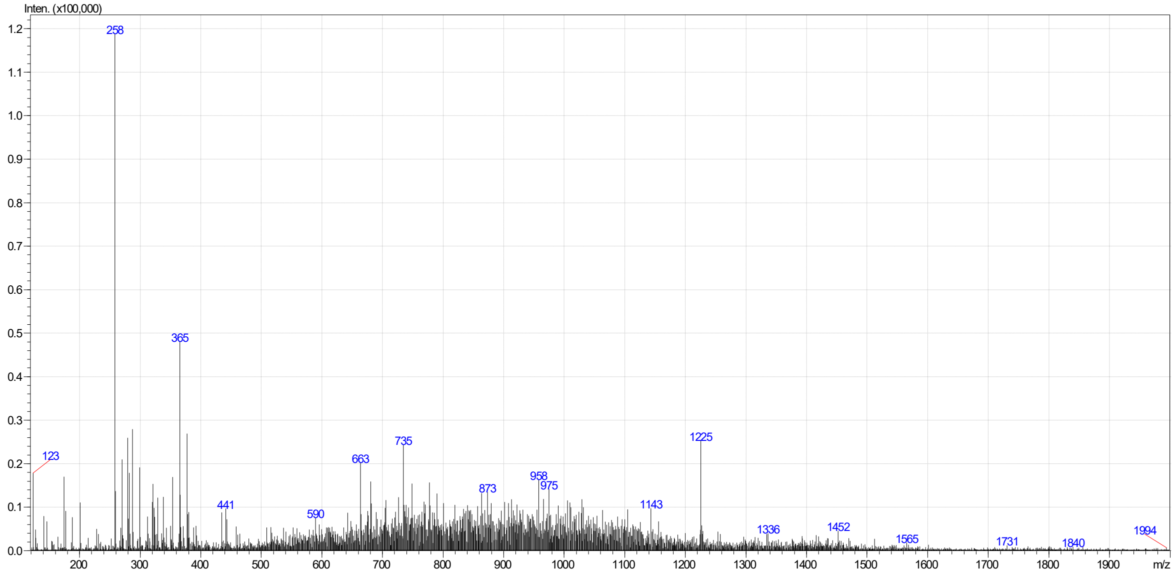

Figure S38. Mass spectra of the fraction (retention time 7.5 to 7.8 min) detected in the sample prepared from encapsulated film containing $FA_{0.83}Cs_{0.17}Pb(I_{0.83}Br_{0.17})_3$ and spiro[TFSI]$_2$ kept at 100 °C for 56 days.

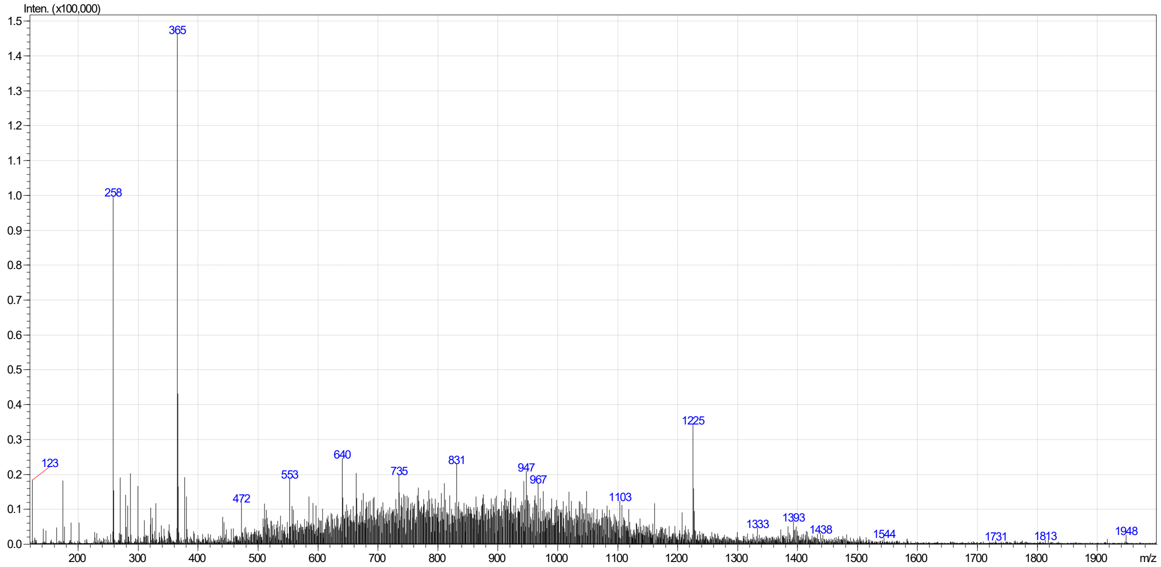

Figure S39. Mass spectra of the fraction (retention time 7.5 to 7.8 min) detected in the sample prepared from encapsulated film containing $MAPbBr_3$ and spiro[TFSI]$_2$ kept at 100 °C for 56 days.

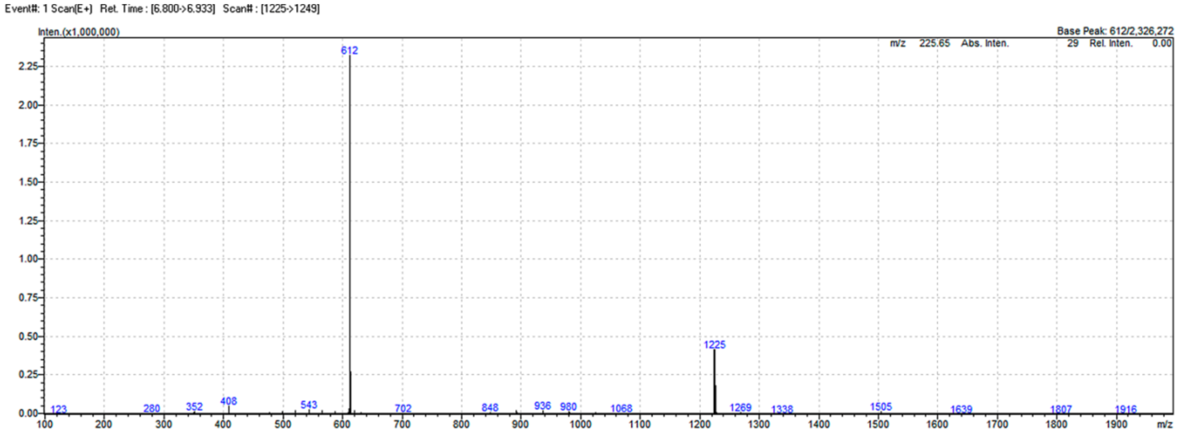

Figure S40. Mass spectra of the first fraction (retention time 6.9 min) detected in the sample prepared from the pure spiro[TFSI]2.

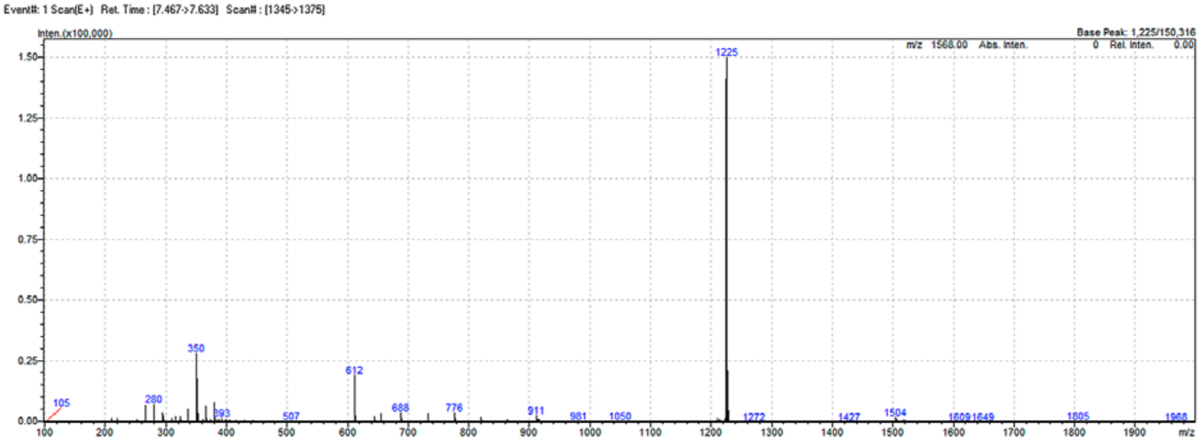

Figure S41. Mass spectra of the second fraction (retention time 7.6 min) detected in the sample prepared from the pure spiro[TFSI]2.

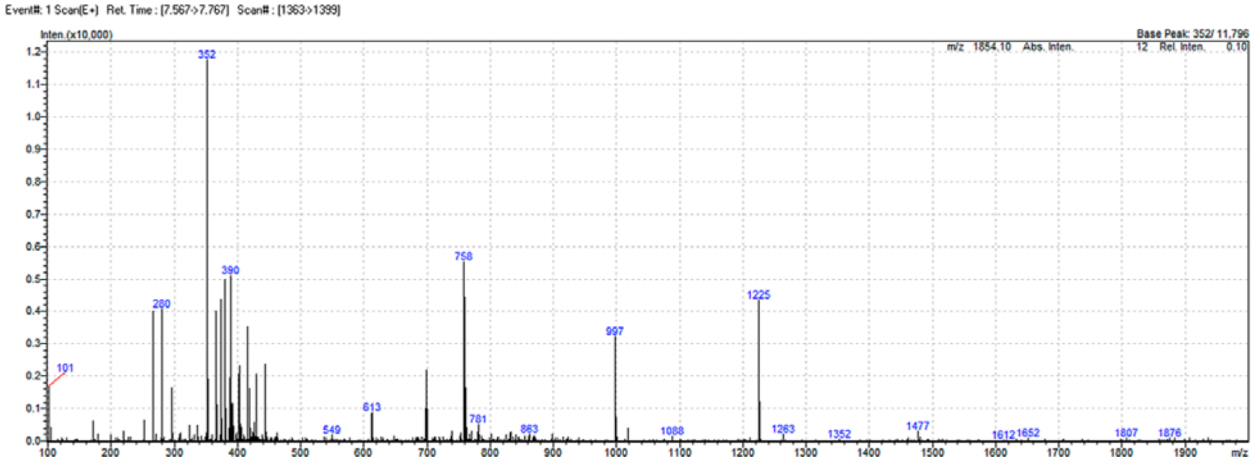

Figure S42. Mass spectra of the pure spiro-OMeTAD (retention time 7.6 min).

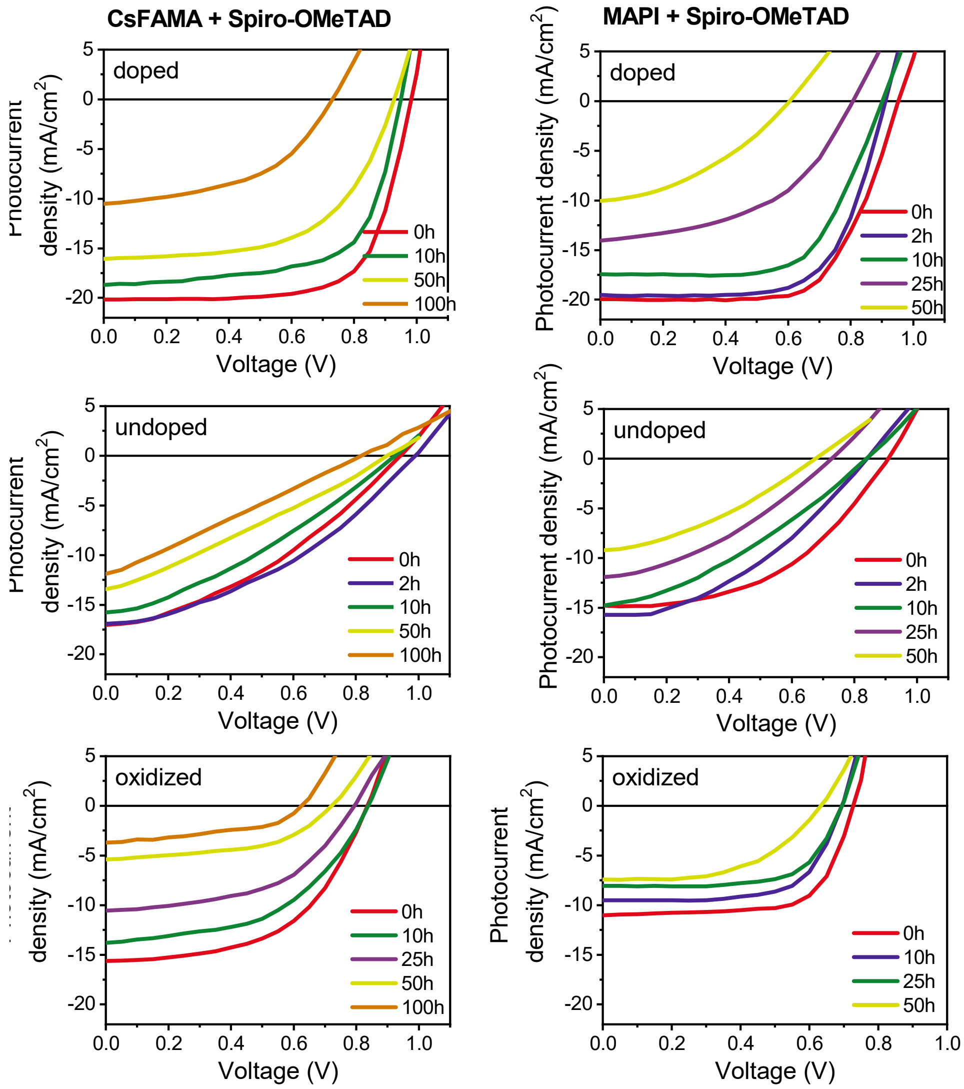

Figure S43. Current-voltage characteristics of the CsFAMA and MAPI perovskite solar cells obtained under ambient conditions at 90 °C.

Table S2. Photovoltaic data of CsFAMA devices based on **doped** spiro-OMeTAD under ambient conditions at 90 °C.

Time	I_{SC}, mA/cm^2	V_{OC}, V	*FF*, %	*Efficiency*, %
0h	20.1	1	68.6	13.8
10h	18.7	0.95	65.2	11.6
50h	16.0	0.93	57.7	8.6
100h	10.5	0.73	48.9	3.75

Table S3. Photovoltaic data of CsFAMA devices based on **undoped** spiro-OMeTAD under ambient conditions at 90 °C.

Time	I_{SC}, mA/cm^2	V_{OC}, V	*FF*, %	*Efficiency*, %
0h	17.0	0.94	33.7	5.9
2h	16.9	0.99	37.9	6.4
10h	15.8	0.92	33.0	4.8
50h	13.4	0.90	28.0	3.4
100h	11.9	0.81	26.1	2.5

Table S4. Photovoltaic data of CsFAMA devices based on **oxidized** spiro-OMeTAD under ambient conditions at 90 °C.

Time	I_{SC}, mA/cm^2	V_{OC}, V	*FF*, %	*Efficiency*, %
0h	15.6	0.84	53.3	6.9
10h	13.8	0.84	50.3	5.8
25h	10.5	0.79	51.4	4.3
50h	5.4	0.73	50.9	2.0
100h	3.7	0.63	44.9	1.1

Table S5. Photovoltaic data of MAPI devices based on **doped** spiro-OMeTAD under ambient conditions at 90 °C.

Time	I_{SC}, mA/cm^2	V_{OC}, V	*FF*, %	*Efficiency*, %
0h	19.9	0.95	66.5	12.6
2h	19.5	0.91	66.6	11.8
10h	17.4	0.90	64.8	10.2
25h	14.1	0.80	48.6	5.5
50h	10.5	0.61	38.0	2.3

Table S6. Photovoltaic data of MAPI devices based on **undoped** spiro-OMeTAD under ambient conditions at 90 °C.

Time	I_{SC}, mA/cm^2	V_{OC}, V	*FF*, %	*Efficiency*, %
0h	14.8	0.90	47.9	6.4
2h	15.7	0.84	39.5	5.2
10h	14.7	0.84	33.6	4.2
25h	11.9	0.73	35.6	3.1
50h	9.2	0.67	35.2	2.2

Table S7. Photovoltaic data of MAPI devices based on **oxidized** spiro-OMeTAD under ambient conditions at 90 °C.

Time	I_{SC}, mA/cm^2	V_{OC}, V	*FF*, %	*Efficiency*, %
0h	11.0	0.73	68.0	5.5
2h	12.1	0.66	67.0	5.4
10h	9.5	0.7	66.7	4.4
25h	8.1	0.69	68.0	3.8
50h	7.4	0,64	52.8	2.5

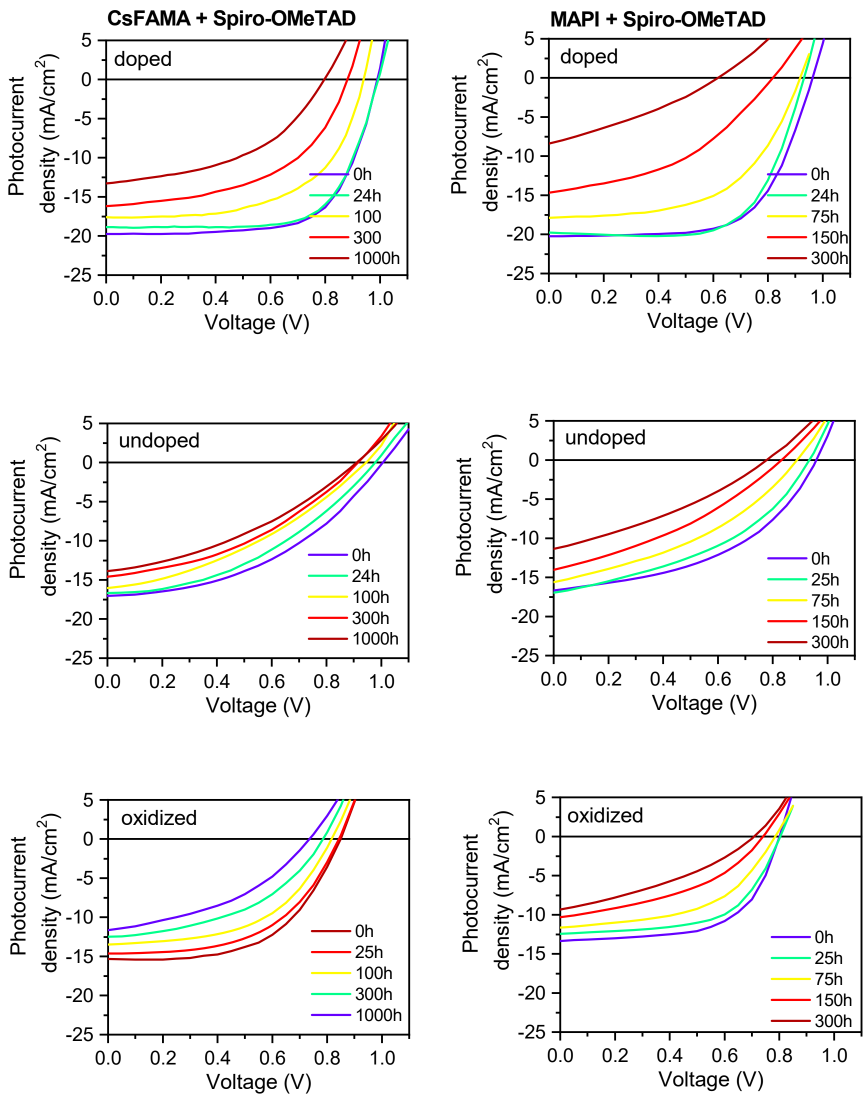

Figure S44. Current-voltage characteristics of the CsFAMA and MAPI perovskite solar cells obtained under nitrogen atmosphere in the dark at 90 °C.

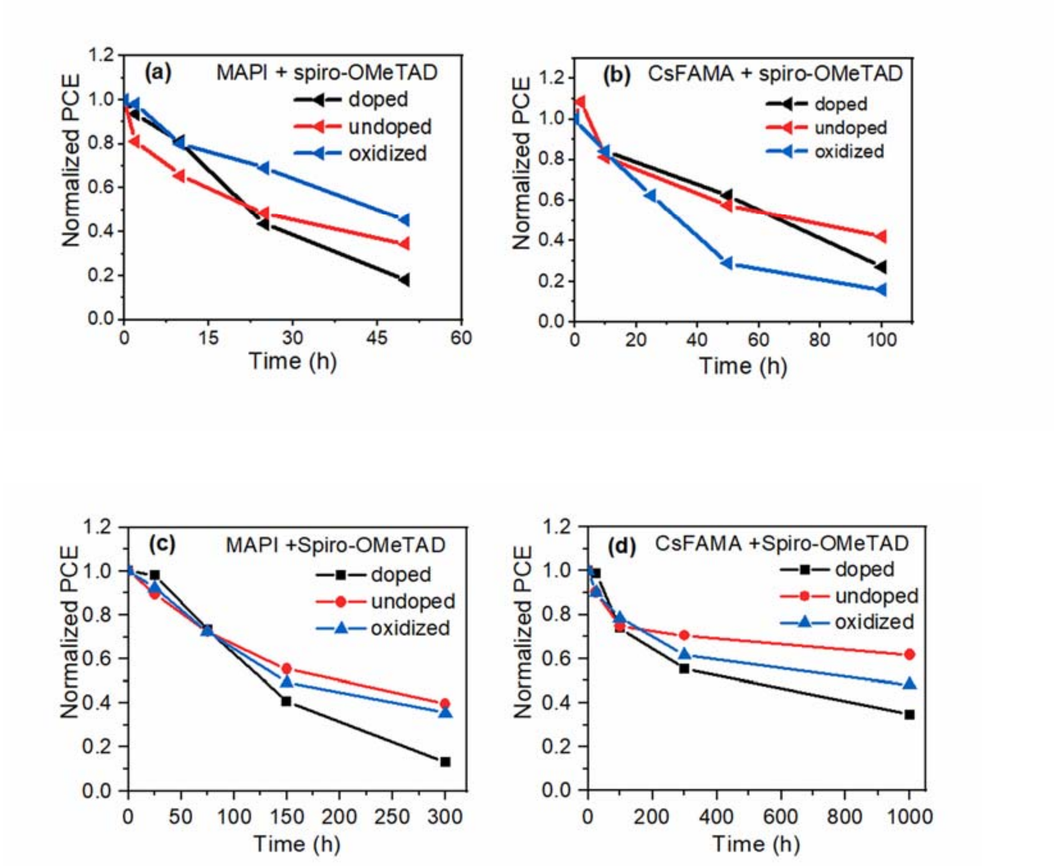

Figure S45. Degradation dynamics of the MAPI and CsMAFA perovskite solar cells with neutral spiro-OMeTAD (doped or undoped) or oxidized spiro-OMeTAD under ambient conditions at 90 °C (a and b) and nitrogen atmosphere in the dark at 90 °C (c and d).

Table S8. Photovoltaic data of CsFAMA devices based on **doped** spiro-OMeTAD under nitrogen atmosphere in the dark at 90 °C

Time	I_{SC}, mA/cm^2	V_{OC}, V	*FF*, %	*Efficiency*, %
0h	19.75	0.99	67.3	13.17
25h	18.87	0.99	69.5	12.98
100h	16.72	0.94	61.9	9.74
300h	16.18	0.88	51.1	7.29
1000h	13.30	0.796	43.0	4.55

Table S9. Photovoltaic data of CsFAMA devices based on **undoped** spiro-OMeTAD under nitrogen atmosphere in the dark at 90 °C

Time	I_{SC}, mA/cm^2	V_{OC}, V	*FF*, %	*Efficiency*, %
0h	17.05	1.02	42.6	7.42
24h	16.69	0.985	40.6	6.68
100h	16.05	0.95	36.3	5.54
300h	14.61	0.91	39.2	5.22
1000h	13.88	0.91	36.3	4.58

Table S10. Photovoltaic data of CsFAMA devices based on **oxidized** spiro-OMeTAD under nitrogen atmosphere in the dark at 90 °C

Time	I_{SC}, mA/cm^2	V_{OC}, V	*FF*, %	*Efficiency*, %
0h	15.32	0.854	56.1	7.34
24h	14.64	0.847	53.3	6.61
100h	13.32	0.81	53.3	5.75
300h	12.48	0.784	46.1	4.51
1000h	11.64	0.784	38.8	3.54

Table S11. Photovoltaic data of MAPI devices based on **doped** spiro-OMeTAD under nitrogen atmosphere in the dark at 90 °C

Time	I_{SC}, mA/cm^2	V_{OC}, V	*FF*, %	*Efficiency*, %
0h	20.24	0.96	64.6	12.56
24h	19.75	0.93	67.2	12.34
75h	17.90	0.91	56.6	9.21
150h	14.69	0.81	43.0	5.12
300h	8.41	0.62	31.1	1.62

Table S12. Photovoltaic data of MAPI devices based on **undoped** spiro-OMeTAD under nitrogen atmosphere in the dark at 90 °C

Time	I_{SC}, mA/cm^2	V_{OC}, V	*FF*, %	*Efficiency*, %
0h	16.68	0.953	46.1	7.33
24h	16.97	0.931	41.5	6.56
75h	15.61	0.89	38.1	5.29
150h	14.05	0.82	35.2	4.06
300h	9.47	0.775	39.5	2.90

Table S13. Photovoltaic data of MAPI devices based on **oxidized** spiro-OMeTAD under nitrogen atmosphere in the dark at 90 °C

Time	I_{SC}, mA/cm^2	V_{OC}, V	*FF*, %	*Efficiency*, %
0h	13.36	0.8	60.5	6.47
24h	12.42	0.8	60.1	5.97
75h	11.63	0.82	49.1	4.68
150h	10.32	0.75	41.2	3.19
300h	9.34	0.701	35.1	2.30

References

(1) Nguyen, W. H.; Bailie, C. D.; Unger, E. L.; McGehee, M. D. Enhancing the Hole-Conductivity of Spiro-OMeTAD without Oxygen or Lithium Salts by Using Spiro(TFSI)2 in Perovskite and Dye-Sensitized Solar Cells. *J. Am. Chem. Soc.* 2014, *136*, 10996–11001.

(2) Giordano, F.; Abate, A.; Correa Baena, J. P.; Saliba, M.; Matsui, T.; Im, S. H.; Zakeeruddin, S. M.; Nazeeruddin, M. K.; Hagfeldt, A.; Graetzel, M. Enhanced electronic properties in mesoporous TiO_2 via lithium doping for high-efficiency perovskite solar cells. *Nat Commun,* 2016, *7*, 10379.

(3) Bi, D.; Tress, W.; Dar, M. I.; Gao, P.; Luo, J.; Renevier, C.; Schenk, K.; Abate, A.; Giordano, F.; Correa Baena, J. P.; Decoppet, J. D.; Zakeeruddin, S. M.; Nazeeruddin, M. K.; Gratzel, M.; Hagfeldt, A. Efficient luminescent solar cells based on tailored mixed-cation perovskites. *Sci Adv,* 2016, *2*, e1501170

(4) Saliba, M.; Matsui, T.; Seo, J. Y.; Domanski, K.; Correa-Baena, J. P.; Nazeeruddin, M. K.; Zakeeruddin, S. M.; Tress, W.; Abate, A.; Hagfeldt, A.; Grätzel, M. Cesium-containing triple cation perovskite solar cells: improved stability, reproducibility and high efficiency. *Energy Environ. Sci.* 2016, *9*, 1989−1997.

(5) Schutt, K.; Nayak, P. K.; Ramadan, A. J.; Wenger, B.; Lin, Y.-H.; Snaith, H. J.; Overcoming Zinc Oxide Interface Instability with a Methylammonium-Free Perovskite for High-Performance Solar Cells. *Adv. Funct. Mater.* 2019, *29*, 1900466.

(6) Aranda, C.; Guerrero, A.; Bisquert, J. Ionic Effect Enhances Light Emission and the Photovoltage of Methylammonium Lead Bromide Perovskite Solar Cells by Reduced Surface Recombination. *ACS Energy Letters* 2019 *4* (3), 741-746

(7) A. Abate, S. Paek, F. Giordano, J.-P. Correa-Baena, M. Saliba, P. Gao, T. Matsui, J. Ko, S. M. Zakeeruddin, K. H. Dahmen, et al., Silolothiophene-Linked Triphenylamines as Stable Hole Transporting Materials for High Efficiency Perovskite Solar Cells, *Energy Environ. Sci.,* 2015, *8*(10), 2946–2953.
